# Ultrasound and neuroinflammation: immune modulation via the heat shock response

**DOI:** 10.7150/thno.96270

**Published:** 2024-05-19

**Authors:** Graham M. Seasons, Carly Pellow, Hedwich F. Kuipers, G. Bruce Pike

**Affiliations:** 1 Hotchkiss Brain Institute, University of Calgary, Alberta, T2N 4N1, Canada.; 2 Department of Clinical Neurosciences, Cumming School of Medicine, University of Calgary, Alberta, T2N 1N4, Canada.; 3 Department of Radiology, Cumming School of Medicine, University of Calgary, Alberta, T2N 1N4, Canada.; 4 Department of Cell Biology & Anatomy, Hotchkiss Brain Institute and Snyder Institute for Chronic Diseases, University of Calgary, Alberta, T2N 1N4, Canada.

## Abstract

Current pharmacological therapeutic approaches targeting chronic inflammation exhibit transient efficacy, often with adverse effects, limiting their widespread use - especially in the context of neuroinflammation. Effective interventions require the consideration of homeostatic function, pathway dysregulation, and pleiotropic effects when evaluating therapeutic targets. Signalling molecules have multiple functions dependent on the immune context, and this complexity results in therapeutics targeting a single signalling molecule often failing in clinical translation. Additionally, the administration of non-physiologic levels of neurotrophic or anti-inflammatory factors can alter endogenous signalling, resulting in unanticipated effects. Exacerbating these challenges, the central nervous system (CNS) is isolated by the blood brain barrier (BBB), restricting the infiltration of many pharmaceutical compounds into the brain tissue. Consequently, there has been marked interest in therapeutic techniques capable of modulating the immune response in a pleiotropic manner; ultrasound remains on this frontier. While ultrasound has been used therapeutically in peripheral tissues - accelerating healing in wounds, bone fractures, and reducing inflammation - it is only recently that it has been applied to the CNS. The transcranial application of low intensity pulsed ultrasound (LIPUS) has successfully mitigated neuroinflammation *in vivo*, in models of neurodegenerative disease across a broad spectrum of ultrasound parameters. To date, the underlying biological effects and signalling pathways modulated by ultrasound are poorly understood, with a diverse array of reported molecules implicated. The distributed nature of the beneficial response to LIPUS implies the involvement of an, as yet, undetermined upstream signalling pathway, homologous to the protective effect of febrile range hyperthermia in chronic inflammation. As such, we review the heat shock response (HSR), a protective signalling pathway activated by thermal and mechanical stress, as the possible upstream regulator of the anti-inflammatory effects of ultrasound.

## 1. Introduction

Inflammation is a natural process that is part of the immune response to stress, pathogens, and general physiological insults. While acute inflammation can be beneficial 1, it can have a deleterious impact if dysregulated 2. Across biological systems, inflammation is a defining and contributing factor of chronic disease, making it a common therapeutic target. To date, very few therapeutics have resulted in successful treatment outcomes for chronic inflammation, or are poorly tolerated and subject to patient comorbidities 3. Additionally, in the case of the central nervous system (CNS), the parenchymal tissue is separated from the vascular system through a complex of endothelial cells connected via tight junctions, maintained by tight junction proteins, called the blood brain barrier (BBB). This barrier prevents pathogens, immune cells, and many small molecules from infiltrating the brain. However, while this is protective in homeostatic conditions, it also prevents therapeutics from accessing the parenchyma, and targeting diseases and disorders of the neurological system. Consequently, treatments that are capable of reaching the brain are of great interest; this has led to increased interest in ultrasound as a method to temporarily disrupt the BBB for the targeted delivery of therapeutics 4. In addition to this, ultrasound is being considered as a means of minimally invasive neurosurgery 5, tumour ablation 6, as well as directly leveraging biological effects on the immune system 7. However, the modulation of the immune system that is induced by ultrasound remains poorly characterized, with no coherent hypothesis linking the upregulation of various disparate anti-inflammatory signalling pathways. A common, highly phylogenetically conserved signalling pathway is the heat shock response (HSR) 8, polysemous in its activation in response to both thermal and mechanical stress, which regulates a broad spectrum of protective signalling pathways essential for cell survival. The HSR is characterized by the upregulation in transcription of HSR-related genes, and changes in HSR-related protein affinity, the most well-known of which are heat shock proteins (HSPs). HSPs are chaperone proteins, responsible for refolding, sequestering, and degrading dysregulated proteins, thereby mitigating toxic conformations and subsequent apoptosis. While one component of a much broader signalling response, HSPs are often treated as a synecdoche for the HSR, used to identify heat shocked cells and demonstrated to have a potent anti-apoptotic effect 9, both in conjunction with, and apart from, the broader signalling response. Traditionally implicated in the protective effect of febrile hyperthermia, recent research has demonstrated a homologous overlap in immune modulation of the HSR beyond with the mechanical effects of ultrasound, possibly constituting a common upstream immune regulator. As a rapidly growing and promising field, it is important to further map and comprehend the cellular signalling pathways driving the beneficial response to ultrasound, as to date, many of the mechanisms remain unclear.

### 1.1 Magnetic resonance guided focused ultrasound

Transcranial high intensity magnetic resonance guided focused ultrasound (MRgFUS) is a minimally invasive surgical technique 5 that can focus mechanical energy deep in the brain, inducing an increase in temperature beyond a lesional threshold at a target location, while sparing the surrounding tissue. MRgFUS procedures are monitored with MR thermometry, which leverages the relationship between temperature and the proton resonance frequency shift 10 to monitor shifts in temperature through changes in phase maps, facilitating real-time thermal imaging. While current applications have focused on essential tremor (ET) 5 and tumour ablation 6 to induce cell death, ultrasound-induced bioeffects extend to the immune system. High intensity focused ultrasound (HIFU) has been suggested to increase tumour cell antigen presentation for targeting by the adaptive immune system, as well as activating endogenous danger signalling pathways 11. Studies on the immune effect of HIFU have largely remained concentrated on cancer and stimulating a T cell response; however, it has also been proposed that HIFU can modulate radiation-induced inflammation and reduce concomitant side effects 12 - a translational result which would be of interest in the treatment of a broad spectrum of inflammatory diseases. Despite this, HIFU remains limited to ablative therapy, with the primary purpose of inducing cell death, making it unsuitable for use in inflamed, but otherwise healthy tissue. The practical translation of ablative MRgFUS procedures to the modulation of inflammation requires the selection of parameters that induce hyperthermia below the physiological limits of tissue damage, analogous to fever, the body's own protective mechanism of hyperthermia. Consequently, while postulated for HIFU, ultrasound-mediated mild hyperthermia instead constitutes a promising and actionable method of ameliorating inflammation across pathologies.

### 1.2 MRgFUS mediated resolution of radiation induced edema

Interestingly, a recent case study detailed a persistent stereotactic radiosurgery (SRS)-related edema, which was resolved after undergoing an MRgFUS thalamotomy 13. In 2015, a 62-year-old man with chronic demyelinating polyneuropathy underwent a SRS thalamotomy to treat tremor, which was transiently successful, regressing to approximately pre-treatment baseline levels of tremor approximately three years following treatment. It is at this time that they were enrolled in a study on MRgFUS thalamotomy. Pre-MRgFUS T2w imaging revealed a large hyperintense region of edema surrounding and extending beyond the SRS target, which was absent from pre-SRS imaging three years prior. SRS is another minimally invasive neurosurgical technique used in the treatment of ET 14, characterized by the focusing of high-dose ionizing radiation to create a targeted brain lesion; as SRS cannot be monitored intra-operatively, lesions can be accompanied by unforeseen radiosurgery related edema, manifesting as a hyperintense zone around the lesion site on T2-weighted (T2w) magnetic resonance (MR) images 13,15,16. Radiosurgery-related edema has been associated with the development of clinical side effects 15, and although no adverse effects were observed in the case study, the T2w hyperintensity exhibited a similar morphology to that associated with inflammation in prior reports 16,17. One-day post-MRgFUS the hyperintensity had both an increased volume and signal intensity on T2w images. However, three months post-MRgFUS the hyperintensity was completely resolved, resembling that of a typical MRgFUS thalamotomy patient 13. Patient medication remained stable leading up to and following the thalamotomy.

**Figure 1:**
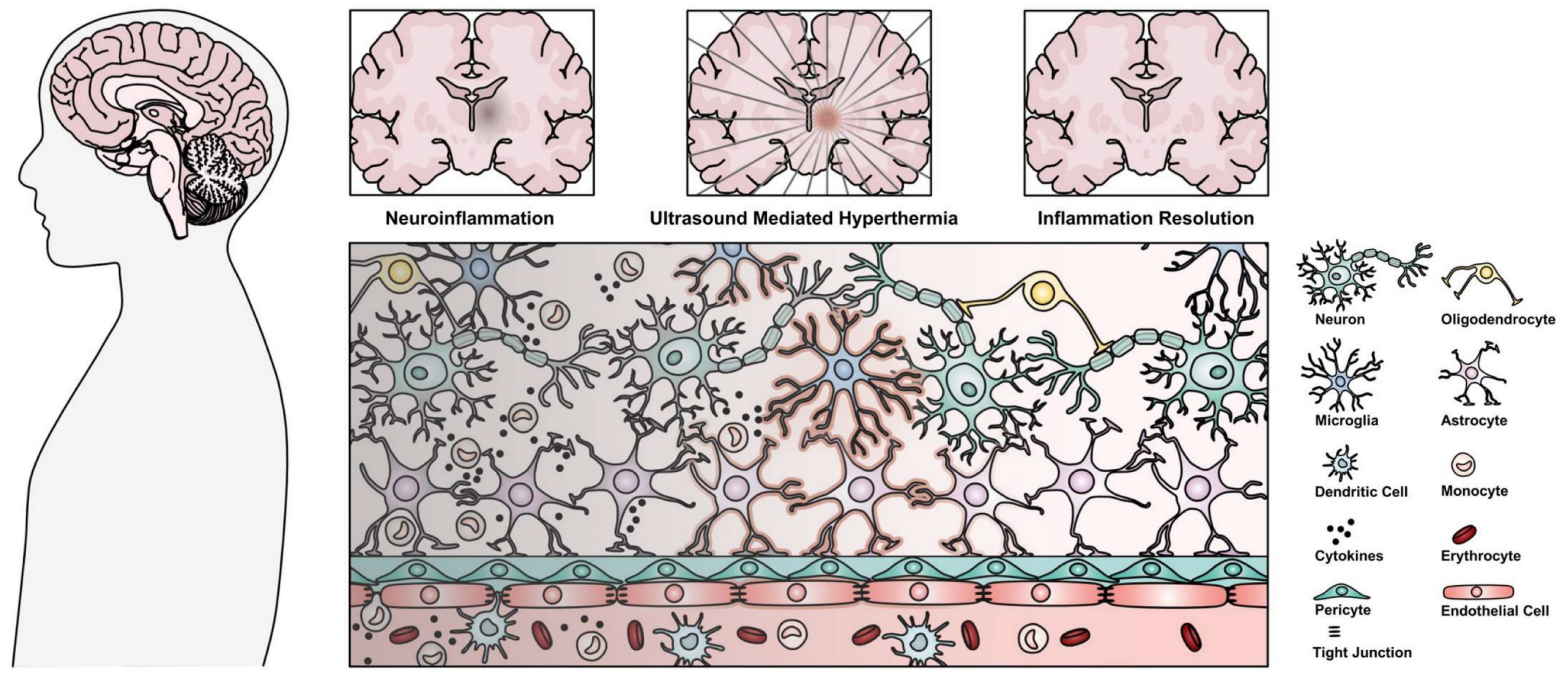
Depiction of a chronic neuroinflammatory lesion located deep in the brain characterized by cellular disruption, and the proposed ability of ultrasound mediated hyperthermia as a method to access and resolve neuroinflammation leading to restored homeostasis. Highlighted cells (microglia, astrocytes, and endothelial cells) are proposed to be stimulated by ultrasound mediated hyperthermia to induce the resolution of neuroinflammation.

While edema cannot be explicitly connected to localized inflammation, radiation is known to induce an inflammatory response in the brain 18, and the resulting T2w hyperintensity has been correlated with long-term macrophage infiltration and inflammation 16,17. Additionally, corticosteroids have been used to treat SRS-related edema 15, indicating a potential inflammatory component to the corresponding imaging finding. As such, the evidence presented in the case study supports the potential of HIFU as a modulator of radiation-induced inflammation 12. Regardless, the biological mechanisms behind the finding remain unclear, and bear further investigation *in vivo*. The following review attempts to cast light on the potential underlying mechanisms behind the described imaging finding, represented in Figure 1, focusing on the ameliorating potential of both the mechanical and thermal effects of ultrasound in chronic inflammation, confluently linked through the HSR.

## 2. The immune response

The immune system is composed of two distinct, but interrelated responses: innate and adaptive, with the innate system present ubiquitously, whereas the adaptive system is bifurcated and present primarily in specialized locations. While the innate immune system non-specifically reacts to and attempts to eliminate any foreign agent, based on molecular patterns, the adaptive immune system is instructed, by the innate immune system, to recognize specific antigens (peptides capable of activating an immune receptor, and initiating a subsequent immune response). For a considerable time, the CNS was considered a site of immune privilege, absent of any native immune cells, and separated from the peripheral immune system due to the impermeability of the BBB 19. The concept of the CNS as an immune privileged site has since changed, more accurately classified as immune restricted, where the direct stimulation of the adaptive response by CNS resident immune cells is very limited 19. Peripheral immune cells stem from a common hematopoietic lineage that differentiates into myeloid and lymphoid cell lineages, which compose the innate, and adaptive immune responses respectively. The CNS lacks direct counterparts of the immune cell subtypes found in the periphery. However, (some of) their roles are observed by glial cells, a subclass of neural support cells, which include oligodendrocytes, astrocytes, and microglia. Microglia and astrocytes primarily constitute the immunocompetent cells of the CNS and can display an array of immune functions, although oligodendrocytes are not bystanders 20. Microglia are considered the resident macrophages of the CNS, stemming from hematopoietic lineage and infiltrating the CNS during development 21, capable of self-renewal, producing and responding to cytokines, phagocytosis, and antigen presentation 22. At rest they are in an immune surveillant state, whereas they are dynamically capable of changing phenotype, both functional and morphological, in response to stimuli. Classically, two microglial phenotypes or activation states have been characterized: M1 and M2, corresponding to an inflammatory and a reparative state, respectively. However, this is an overly simplistic view 22, as microglia can co-express both pro- and anti-inflammatory markers used for characterization. Moreover, depending on the stimulatory ligand *in vitro*, the morphological phenotype and behaviour of microglia is altered 23, exhibiting varied phagocytosis, migration, and transcriptomic profiles between colloquially described M1 states, indicating a more complex functional heterogeneity than a simple paradigm of binary activation. Alternately, astrocytes are derived from neuroepithelial stem cells, and in a homeostatic environment are involved in maintaining proper BBB integrity, monitoring the cellular environment, and supporting other CNS cells 24. Similar to microglia, astrocytes are also involved in cytokine production and detection, as well as phagocytosis, albeit on a reduced scale 19. Astrocytes are also colloquially accepted to adopt both pro- and anti-inflammatory states in response to stimuli - termed reactive astrogliosis, using A1 and A2 to denote each respectively 25. As with microglia, the phenotypic activation states of astrocyte are likely more plastic than this proposed dichotomy, corresponding to heterogeneous populations of activation states 26.

Lymphocytes, of which T cells are an important subset, are the dominant actors in executing the adaptive immune response. The adaptive response is targeted to a specific antigen that is presented in the context of the major histocompatibility complex (MHC) by antigen presenting cells (APCs). The innate and adaptive immune systems act in a synergistic fashion, where innate immune cells act as APCs to activate and instruct adaptive immune cells. Exogenous antigens are processed following phagocytosis of the foreign agent by APCs and displayed through MHC II for recognition by cluster of differentiation four positive (CD4+) T cells 27. Proper activation of T cells requires efficient coordination of cell-to-cell interactions between APCs and T cells. Within the peripheral immune system, the dominant APCs are dendritic cells (DCs), monocytes, and macrophages. After phagocytosing antigens at sites of infiltration of foreign agents, APCs traffic via the lymphatic system to secondary lymphoid tissues, which allow for the localized interaction of APCs and a dense population of lymphocytes, which subsequently facilitates the egress of stimulated, antigen-specific, T cells into circulation, enabling the recognition and destruction of pathogenic cells, and instruction of the local immune response 27. In order to assure proper recognition of presented non-self antigens, and prevent excessive recognition of self antigens, T cells are selected in a 2-step process during development. In the thymic cortex, they are first tested against a specialized section of the cortical epithelium for (self-)MHC recognition, and subsequently for autoreactivity of self-tissue proteins in the thymic medulla 28; cells that fail either stage undergo apoptosis, ensuring proper self/non-self differentiation. In parallel with the recognition of exogenous antigens by CD4+ T cells, endogenous, as well as intracellular foreign antigens are displayed by MHC I for detection by CD8+ T cells, a mechanism which is not restricted to professional APCs - this generally occurs in response to a viral infection 27.

While neither microglia nor astrocytes can directly initiate an adaptive immune response, there is cross-communication between the CNS and the peripheral immune system and both cell types are thought to be able to re-activate peripherally initiated T cell responses. Both astrocytes and microglia are capable of presenting antigens, as they are able to express both MHC I and II 29,30. However, they are not considered professional APCs in the classical sense, because they lack high expression of costimulatory molecules. In addition, they can produce various cytokines that affect the local inflammatory milieu, supporting or inhibiting T cell responses. There are several additional mechanisms by which peripheral and CNS inflammatory responses can affect each other. Within the CNS, glial and neuronal cells release extracellular vesicles (EVs) which can communicate cellular state, and transfer cytokines, proteins, and microRNAs (miRNAs) between cells. The contents of these EVs correspond to the source cell, and its reactive or degenerative state 31. EVs have gained interest in recent years as a therapeutic target, as they are implicated in a number of neurodegenerative diseases and processes 31, and are capable of crossing the BBB 32, leading to proposals of using EVs as diagnostic markers 33. In crossing the BBB, EVs can also transfer inflammation from the CNS to the periphery; following an intracerebral injection of interleukin-1β (IL-1β) in a mouse model, astrocyte-derived EVs were found circulating in the blood stream and inducing an inflammatory response in the liver, as well as leading to leukocyte infiltration in the brain 32. As such, while the CNS immune cells do not directly initiate an adaptive immune response via antigen presentation, they can induce leukocyte infiltration through the dispersion of pro-inflammatory EVs and subsequently act as APCs in a localized reactivation of an adaptive immune response. To aid this process, while usually leukocyte trafficking across the BBB is limited, its integrity becomes compromised during inflammation or injury, allowing infiltration of various peripheral immune cell types 19,34. While implicated in facilitating the activation of the adaptive immune system from the CNS, the permeability of the BBB to EVs also affords opportunity for novel therapeutics, with proposals of using EVs for drug delivery to deep brain structures 33.

### 2.1 Peripheral inflammation translocation to CNS

Peripheral inflammation can also transfer to the CNS, independent of immune cell infiltration. In the innate immune system there are a number of receptors that recognize damage or pathogen associated molecular patterns (DAMP/PAMPs), inducing an inherent, immediate inflammatory response 27. While many different types of immune receptors recognize DAMP/PAMPs, classified as pattern recognition receptors (PRR), the toll like receptors (TLRs) remain one of the most well studied and characterized families, and are capable of responding to a wide variety of both exogenous and endogenous stimulatory molecules 35. This genetically engrained response allows for the rapid mobilization of immune cells in an attempt to eliminate and control the deleterious stimulus; this casts a broad net, responding to a type of immune challenge through the release of cytokines and chemokines 27. The innate immune response is not pathogen specific, with different TLRs instead recognizing classes of DAMPs/PAMPs - these TLRs can be segregated into two groups: cell membrane, and intracellular receptors. Cell membrane receptors (TLR1, TLR2, TLR4, TLR5, TLR6, TLR10) respond to bacteria, proteins, lipids, and lipoproteins, while the intracellular compartmental receptors (TLR3, TLR7, TLR8, TLR9) respond to nucleic acids (DNA, RNA, miRNA) 36; the TLR response is highly conserved across species and cell types, although the specific receptors expressed are not 36, with more TLRs expressed in mice than humans.

Upon activation by an appropriate ligand, an adaptor protein is recruited to the cytoplasmic Toll/IL-1 receptor (TIR) domain of the TLR 37, which controls the subsequent intracellular signalling pathway activated. TLRs 1, 2, 4, and 6 all use the myeloid differentiation primary response 88 (MyD88) adaptor like (MAL) adaptor protein, which recruits MyD88 and subsequently activates a signalling cascade through the interleukin-1 receptor-associated kinases (IRAKs), and tumour necrosis factor receptor-associated factor 6 (TRAF6); this activates downstream c-Jun N-terminal kinase (JNK) and nuclear factor kappa B (NF-κB) inflammatory pathways 35. NF-κB is bound in the cytoplasm by inhibitor of NF-κB (IκB), which is degraded through phosphorylation by the IκB kinase (IKK) complex, resulting in the nuclear translocation of NF-κB, and the subsequently induced transcription of tumour necrosis factor alpha (TNFα), IL-1β, and IL-6 35. All TLRs which use either MAL/MyD88 or MyD88 adaptor proteins (TLR1, TLR2, TLR4-10) utilize this signalling pathway. In addition, intracellular TLRs 7-9 use a direct signalling pathway through TRAF6, which leads to the nuclear translocation of interferon regulatory factor 7 (IRF7) 35, and the induction of interferon alpha (IFNα) 38. While MyD88 is involved in most TLR signalling, the TIR domain-containing adaptor-inducing interferon beta (TRIF) is used by both TLR4, and TLR3, and activation of this pathway results in NF-κB nuclear translocation and IFNβ production via IRF3 35.

Peripheral TLR activation can result in neuroinflammation. In a murine model of sepsis, induced by an intraperitoneal injection of 5 mg/kg of lipopolysaccharide (LPS, a TLR4 ligand) 34,39,40, persistent neuroinflammation was observed 10 months post-injection, while peripheral inflammation was resolved within 7 days 39. While LPS is used as a model of sepsis due to its ability to induce severe, widespread peripheral inflammation, it can also act as a model of more general peripheral infection, especially when investigating neuroinflammation. The persistent neuroinflammation was characterized by an elevated level of TNFα in the brain parenchyma, with protein levels observed at 24 hours post-injection sustained at 10 months. In a TNFα receptor (TNFR1/2) knockout mouse, peripheral LPS injection did not result in elevated TNFα levels in the brain 39, indicating the TNF signalling pathway is responsible for translocation of inflammation into the CNS, and is widely induced upon activation of any TLR. As such, it acts as a model of chronic sterile CNS inflammation.

**Figure 2:**
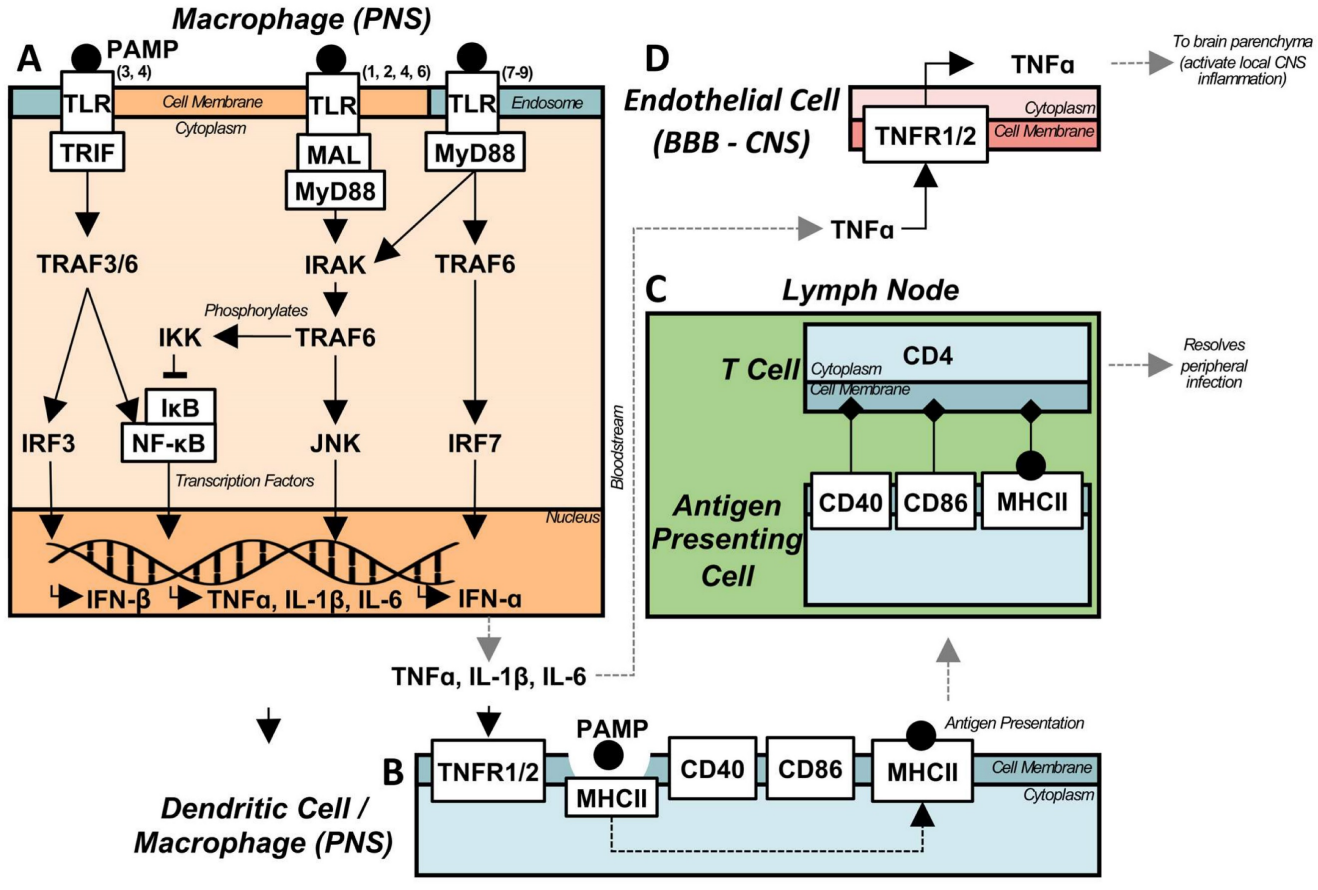
Acute peripheral infection can translocate to the CNS and cause chronic neuroinflammation. *(A)* Depiction of TLR signalling for both the intracellular, endosomal TLRs (3, 7-9) and the cell surface receptors (1, 2, 4-10). During an infection, PAMPs are recognized by TLRs, causing cytoplasmic signalling adaptors to associate with their respective, activated, TLR. The majority of TLRs utilize MyD88, either directly (TLRs 5, 7-10), or via an additional adaptor protein MAL (TLRs 1, 2, 4, 6). However, TLRs 3 and 4 utilize a MyD88-indepenent pathway via TRIF. The majority of TLR and MyD88 signalling is facilitated by IRAK, which subsequently activates TRAF6 leading to downstream NF-kB nuclear translocation, through IKK phosphorylation of IkB, and JNK pathway activation leading to pro-inflammatory cytokine transcription. Alternately, the endosomal TLRs are capable of producing type I interferons through the activation of IRF7. The MyD88-independent pathway activates TRAF3/6 through TRIF, leading to both NF-kB and IRF3 activation, again leading to pro-inflammatory cytokine and type I interferon transcription. *(B)* Following the peripheral activation of immune cells by DAMPs, and the subsequent production of inflammatory cytokines, APCs respond to the pro-inflammatory environment, and become activated, expressing co-stimulatory molecules necessary for activating the adaptive immune response. PAMPs are phagocytosed, and processed for display by MHC II, after which the activated APC traffics to lymphatic tissue. *(C)* The APC binds to the respective T cell receptors, activating the pathogen specific, adaptive immune response. Activated lymphocytes then traffic to infected tissue and target infected cells to assist in the resolution of peripheral infection. *(D)* Pro-inflammatory cytokines produced from peripheral infection, both circulating and generated by circulating monocytes, are capable of activating immune receptors on the endothelial cells of the BBB. Specifically, TNFa activates its receptors, leading to the production of TNFa within the parenchyma, activating pro-inflammatory cytokine production and positive feedback loops, eventually leading to chronic, sterile neuroinflammation.

While the LPS model was first described to induce holistic brain inflammation, with early timepoints following stimulation resulting in inflammation of the substantia nigra (SN), hippocampus, and cortex 39, further research has demonstrated preferential, localized, chronic inflammation. TNFα messenger RNA (mRNA) levels are significantly elevated in the hippocampus and midbrain 1 month post-LPS compared to control, while this is not observed in the cortex or striatum 40. It has been proposed that the regional effects induced by peripheral inflammation are mediated by microglia numbers 39, as well as anti-inflammatory signalling pathway component (i.e. CD200/CD200R) concentrations 40. Despite the relatively simple transfer of peripherally induced LPS inflammation to the CNS via a single cytokine (Figure 2), the etiology of the induced chronic inflammatory state is less clear.

### 2.2 Heterogeneity of PRR activation

Traditionally, research into neurodegenerative disease has focused on the mechanisms behind the initiation of an inflammatory, or neurodegenerative state, and the corresponding ligands or molecules involved in this initiation. This can be restrictive in developing a broad understanding of the progression of inflammation, as the paradigm of a specific PRR activation inducing a characterized result does not always account for the downstream inflammatory cascade *in vivo*, or the larger environmental context. Ethanol and amyloid beta (Aβ) are both characterized as TLR4 41, and TLR2/4 35 ligands respectively, and both receptors are implicated in a wide spectrum of neurodegenerative diseases 42. However, they do not solely induce TLR2/4 stimulation. Recently, transgenic Aβ overexpression mouse models displayed inflammation, and demonstrated increased microglial reactivity surrounding nucleic acid positive Aβ clusters, and concomitant increase in type-I IFN production and IRF7 transcription, indicating likely intracellular compartmental TLR signalling. Blocking downstream IFN signalling by inhibiting type-I IFN receptors (IFNAR) improved pathology, indicating the necessity of considering all components contributing to the perpetuation of an inflammatory cascade 43.

In a model of chronic ethanol-induced neurotoxicity, ethanol induced the release of microglial EVs containing the miRNA let-7b and high mobility group box protein 1 (HMGB1), which was found to stimulate TLR7 and induce IFNα, TNFα, and IL-1β production in a rat hippocampal-entorhinal slice culture; blockade of TLR7 reduced the activation of the MyD88 signalling pathway 44. While TLR4 blockade prior to ethanol administration will eliminate the inflammatory response 41, during chronic ethanol exposure endogenous TLR ligands are released, expanding the inflammatory purview beyond a single TLR. In Alzheimer's disease (AD) patients a similar endogenous signalling pathway is induced, characterized by increased levels of let-7b in the cerebral spinal fluid (CSF) 45, although it should be noted that the pro-inflammatory effect of let-7b and miRNAs in general is dependent on environmental, or EV context 44,46. While there has been a focus on let-7b as an endogenous TLR7 ligand, many other miRNAs have the ability to activate TLR7, including miRNA-21 47,48, which is upregulated by HMGB1 47 and overexpressed in inflammation 48. Consequently, the confluent downstream activation of TLRs by induced endogenous ligands should be considered when investigating the *in vivo* effect of an exogenous ligand.

Furthermore, TLRs act in a synergistic fashion. TLR4 stimulation has been shown to lead to the endosomal localization of TLR7, resulting in increased expression 49 and co-stimulation of receptors that can amplify the inflammatory response 50. As the presumed method of translocation of peripheral inflammation into the CNS, it is important to understand the TNFα signalling cascade in inflammation. Through the TNFR receptors, TNFα induces a positive, pro-inflammatory feedback loop, where increased TNFα levels increase the transcription of the cytokine. Additionally, TNFα has been shown to induce reactive oxygen species (ROS), HMGB1 51 (a TLR4 ligand), type-I IFN in an IFNAR-mediated positive feedback loop 52, and an upregulation in miRNA release. Additionally, downstream effects of TNFα signalling include the activation and priming of different PRR responses, such as that mediated by the nucleotide-binding oligomerization domain (NOD)-like receptor family pyrin domain containing 3 (NLRP3) inflammasome, a cytosolic complex which potently induces IL-1β, and caspase-1 53. The diversity in pathways upregulated in inflammation necessitates the consideration of both the local, cellular context, and that of the larger environment.

### 2.3 Sex differences

Mirroring the heterogeneity in the signalling pathways that are activated in inflammation, the immune response varies with sex 54, age 55, and the circadian rhythm 56. In early postnatal development, female-derived (FD) murine microglia exhibit increased triggering receptor expressed on myeloid-2 (TREM2) expression, associated with increased phagocytosis, as well as a less reactive phenotype when compared to male-derived (MD) cells 57. In adult mice, FD microglia display a neuroprotective or reparative phenotype 57, which is sustained following transplantation into male mice, resulting in reduced lesion volume in a model of ischemic stroke 58. However, in response to DAMP/PAMPs the response of FD immune cells is more robust 54,57,59, which can lead to improved pathogen clearance 54,59 and an increased susceptibility to chronic inflammation and autoimmunity, especially in aged populations 54,55. Immune cell phenotype is modulated by the expression of sex hormones in a dose dependent fashion, where high levels of endogenous estrogen contribute to a reparative phenotype in microglia, astrocytes, monocytes, and T cells which is lost with age 54,57,60, potentially contributing to the increased female bias of many autoimmune and neurodegenerative diseases.

In a mouse model of AD, peripheral immune cell infiltration was greater in 12-month-old female mice compared to their male counterparts 61. Endothelial nitric oxide synthase (eNOS) is an important mediator of vascular health and cerebral blood flow (CBF) 62, and its impairment has been associated with increased BBB permeability and leukocyte infiltration into the CNS 63, as well as concomitantly increased inflammation 64. In young animals, FD endothelial cells express more eNOS 65, which exhibits a symbiotic relationship with estrogen (E2; 17β-estradiol) 66 via activation of estrogen receptor α (ERα) 67, leading to a decreased risk for cardiovascular disease 68 - conversely, a reduction of estrogen with age results in decreased eNOS and increased risk for cardiovascular disease 68, as well as an exacerbated inflammatory response 54,69. While the relationship between low estrogen levels and increased inflammation has been characterized in humans 54, mouse estrogen is largely constant over the lifespan 70, with age instead correlating with a decrease in ERα expression in bone marrow derived macrophages 69. As eNOS is directly modulated by E2 through ERα 67, decreased estrogen signalling in both middle aged mice and humans, leads to a loss of eNOS-mediated neuroprotection with age, and potentially exacerbating inflammation. Contextualizing these results, a broad array of endogenous factors potentially contributing to sex differences in the immune system exist, in addition to changes with age: increased type-I IFN production occurs in the ageing brain, reducing the production of brain derived neurotrophic factor (BDNF), a neurotrophin with anti-inflammatory properties 71 downstream of eNOS 72, from the choroid plexus 73.

Aside from the influence of sex hormones, there are chromosomally mediated sex differences in immune responses. Due to the presence of two X chromosomes, there are multiple copies of the same gene present in the genome of female immune cells. Typically, these doubly present genes are subject to X chromosome inactivation, a process by which one of the X chromosomes is inactivated, leading to comparable mRNA and protein expression levels as those in male cells 59. However, this process is incomplete, and distally located genes can escape inactivation. In the immune system this is of consequence, as TLR7 is distally located on the X chromosome, and can escape inactivation 59. This partially explains the increased production of IFNα in response to viral infections 59, and the increased female bias of autoimmune interferonopathies such as systemic lupus erythematosus 54,74. Needless to say, chronic inflammation is pathologically complex, with an intricate web of contributing endogenous and exogenous factors - this complexity necessitates the development of therapeutic methods targeting a broad spectrum of pathways.

## 3. Ultrasound and inflammation - mechanical mechanisms of resolution

HIFU ablation of tissue involves the confluence of multiple biophysical mechanisms including hyperthermia, and mechanical stimulation, which each have immunomodulatory potential. Much of this research has concentrated on cancer model systems 11, where the desired outcome is the destruction or disruption of pathological tissue, complicating the direct translation of the signalling pathways involved into other etiological contexts where ablation or lesioning is not a desired outcome. Consequently, the bulk of immunological studies concentrating on the effect of ultrasound on neurodegenerative diseases and inflammatory conditions have used LIPUS. LIPUS is a heterogeneous term, encompassing a wide variety of ultrasound parameters, a number of which have been elucidated in Table 1. Typical parameters reported are: spatial peak temporal average intensity (*I_SPTA_*) of 10 to 528 mW/cm^2^, centre frequency (*f_C_*) of 1 to 1.875 MHz, pulse repetition frequency (*f_R_*) of 1 Hz to 6 kHz, pulse burst length of 17 μs to 0.2 s, and individual treatment duration ranging from 5 to 20 minutes *in vivo*, and up to several hours *in vitro*. Comparatively, HIFU is intended for ablation, with typical parameters of: 0.5 to 8 MHz, acoustic power ranging from 100-10,000 W/cm^2^, with short continuous wave sonication times of 10-25s, and focal temperature increases in excess of 50-60 ^o^C 5,6. While high intensity therapeutic ultrasound has been previously defined at an intensity of ≥ 3W/cm^2^ 75 for therapeutic application in an *in vitro* model of inflammation 76 (Table 1), studies at this intensity remain sparse. Essentially, LIPUS uses parameters at or below those used in clinical diagnostic ultrasound 75 to apply a mechanical force to local cells in the targeted area, including resident neurons, glia, and infiltrating immune cells, thereby activating pathways which modulate the inflammatory milieu and immune response, while HIFU uses intensities orders of magnitude higher for the purpose of thermal ablation. Investigations into LIPUS as a treatment for chronic inflammation have demonstrated promising results, in both the CNS and a diverse array of peripheral tissues. To date, the mechanisms behind the mitigating effect of LIPUS on inflammation are poorly understood, as studies are conducted in a wide variety of tissues with heterogeneous ultrasound parameters and disease states. Developing a holistic understanding thus requires the integration of information from numerous disparate studies (see Table 1). Recent *in vivo* studies have investigated the efficacy of LIPUS in neurodegenerative disease and models of cerebral trauma, with beneficial results consistently observed. While there is a consistent finding of upregulated BDNF 64,77-80 corresponding to decreased inflammation with LIPUS application in inflammatory models, the underlying mechanisms are unclear.

### 3.1 Brain derived neurotrophic factor

Several mechanisms have been proposed to underly LIPUS mediated upregulation of BDNF, through *in vitro* studies. The application of LIPUS to BV-2 microglial cells led to the upregulation of BDNF, along with phosphorylated cyclic adenosine monophosphate response element-binding protein (p-CREB), phosphorylated protein kinase B (p-Akt), and phosphorylated tyrosine protein kinase B (p-TrkB), both in LPS and control conditions; with LPS administration, LIPUS additionally downregulated pro-inflammatory cytokines, TLR4, MyD88, and downstream kinase and NF-κB transcription 81. TrkB and BDNF engage in a positive feedback loop, with BDNF acting as a ligand for TrkB, subsequently activating the downstream signalling factor Akt, and CREB, upregulation of which is associated with increased BDNF production - both Akt and CREB were required for BDNF expression in response to LIPUS 81. Akt is a typically anti-inflammatory signalling molecule, the activation of which has been demonstrated to reduce inflammation in an LPS model of sepsis 82. Within the intracellular context of LPS-stimulated microglia, Akt is proposed to increase CREB expression, in addition to directly inhibiting the phosphorylation of JNK, and the nuclear translocation of NF-κB 83. A similar upregulation of BDNF in cultured astrocytes was observed 81 in response to LIPUS, although the mechanism of action is likely different 84. LIPUS stimulated BDNF production by naïve astrocytes is thought to be induced by increased Ca^2+^ influx, and calcium kinase signalling pathways. Interestingly, while this pathway again relies on Akt, CREB is downregulated in response to LIPUS in astrocytes, indicating the existence of a distinct pathway from their microglial counterpart. This difference is emphasized by the increased nuclear translocation of NF-κB, a pro-inflammatory transcription factor, inhibition of which led to the elimination of LIPUS-mediated BDNF production 84. Furthermore, a similar Ca^2+^ dependent mechanism is thought to be responsible for the confluent increase of BDNF with neuronal activity 85, potentially indicating parallel signalling pathways and co-interaction of neurons and astrocytes in BDNF production, beyond the BDNF/TrkB positive feedback loop, as a neuromodulatory process. Increased BDNF has been demonstrated in response to LIPUS in a model of cuprizone-induced demyelination, and chronic restraint stress, as well as accelerated remyelination, a process attributed to neuromodulation 78. In considering LIPUS mediated neuromodulation *in vivo*, changes in local BDNF and neuronal activity cannot be divorced from the influence of surrounding glial cells.

It is important to note that while studying the astrocytic origin of BDNF production, an inflammatory challenge prior to LIPUS stimulation was not used, as described in the study by Liu *et al.* mentioned previously 84. *In vivo* LIPUS treatment of mice resulted in increases in BDNF, CREB, NF-κB, and TLR4, with no significant increase in pro-inflammatory cytokines. However, after an LPS challenge, LIPUS treatment resulted in decreased TLR4 and NF-κB activation, while maintaining the upregulation of BDNF and CREB 79. This could indicate that the induction of separate signalling pathways depends on the context of the glial phenotype, cellular environment, and the local cell populations, or that microglial BDNF production through the TrkB/Akt/CREB pathway is dominant in the overall effect of LIPUS. Regardless of the cell of origin, or mechanism of production, LIPUS treatment both *in vitro* and *in vivo* modulates the endogenous release of neurotrophic factors associated with the reduction of inflammation, constituting one potential branch of a therapeutic pathway, while avoiding potential unwanted side effects 86.

### 3.2 Blood brain barrier

Coincident with the release of neurotrophic factors, LIPUS has been observed to reduce peripheral immune cell infiltration, and increase the stability of the BBB 77,80. Peripheral immune cell infiltration and BBB disruption are implicated in the etiology and exacerbation of neurodegenerative diseases 87,88, aging 88, and in the progression of vascular dementia (VaD) and AD, as pro-inflammatory cytokines reduce the expression of tight junction proteins integral to maintaining BBB function 77. In a model of traumatic brain injury (TBI), LIPUS was applied immediately, and daily thereafter, which resulted in a decrease in reactive microglia, infiltrating neutrophils, metalloproteinase 9 (MMP-9) expression, and an increase in forkhead box protein O1 (FOXO1) expression 80. MMP-9 is associated with increased cleavage of tight junctions and disruption of endothelial cells, and its upregulation is associated with the development of edema 89; alternately, FOXO1 is a downstream factor of the Akt pathway 90, and is associated with reduced apoptosis of endothelial cells and preventing tight junction loss, corresponding to reduced BBB permeability 91. In combination, the effects of LIPUS led to decreased long term neurological deficits and edema. However, this study applied LIPUS longitudinally, starting in an acute stage from the induction of injury, an immediacy in treatment that is not available in chronic inflammatory diseases. The 6-hydroxodopamine (6-OHDA) model of Parkinson's disease (PD) exhibits BBB disruption, and has been used in the investigation of LIPUS on BBB stability in the chronic stage 77. Starting LIPUS treatments two weeks following intracerebral 6-OHDA injection, and continuing for the next six weeks, resulted in a reduction of glial cell reactivity and pro-inflammatory cytokine expression, while the expression of tight junction proteins zonula occludens-1 (ZO-1) and claudin-5 were increased 77. Interestingly, BDNF was not significantly increased in this model, potentially indicating the dominant therapeutic factor was tight junction protein related; it should be noted that glial cell line-derived neurotrophic factor (GDNF) was significantly upregulated following LIPUS in this model, which is neuroprotective and linked to the upregulation of claudin-5 92.

Endothelial cells are integral to maintaining proper vascular and BBB function, as well as secreting factors to maintain homeostasis, such as eNOS. Recently, eNOS has been found to be essential to the beneficial effect of LIPUS in a model of VaD, reducing white matter lesions, increasing neurotrophic factors, improving cognitive function and CBF; these benefits were abolished in an eNOS knockout mouse 64. eNOS is bound to caveolin-1 (Cav-1) in the caveolae of endothelial cells, and is released upon mechanical stimulation 93 through the aid of a heat shock protein 90 (HSP90) and Akt dependent process 94. Furthermore, caveolae and Cav-1 is increased in response to mechanical or thermal stimulation 93, potentially corresponding to a concomitant increase in eNOS. While an eNOS knockout could not be generated for the 5xFAD transgenic AD mouse model, there was similar correlation between the LIPUS-induced upregulation of eNOS, and the production of BDNF, phagocytosis of Aβ, improved cognitive performance and CBF in this model, which was also accompanied by an increase in HSP90 64.

Pharmaceutically, sildenafil, an inhibitor of phosphodiesterase-5 used commonly to treat erectile dysfunction, has many of the same neuroprotective benefits as those observed with LIPUS, and acts as a parallel from which to deduce potential underlying pathways. Mechanistically, in a model of experimental autoimmune encephalomyelitis (EAE), sildenafil functioned by decreasing inducible NOS (iNOS), a pro-inflammatory species, while increasing the expression of eNOS 72,95. Sildenafil increases the expression of adenosine monophosphate (AMP)-activated protein kinase (AMPK), which signals in a positive feedback loop with eNOS, where AMPK activates eNOS, and eNOS produced NO again increases AMPK activation 72. Furthermore, AMPK decreases mammalian target of rapamycin (mTOR) expression, resulting in the upregulation of CREB and BDNF 72. In addition to the generation of neurotrophic factors, the eNOS/AMPK pathway is associated with increased autophagy activity 72, a process dysregulated in aging, neurodegenerative diseases, and inflammation 96; however, as autophagy was measured using absolute autophagy related protein (microtubule-associated protein 1A/1B-light chain 3, beclin-1, autophagy related 5) levels, instead of autophagic flux 96, a definitive statement on the influence of this pathway on autophagy cannot be made. The eNOS/AMPK axis is a potent anti-inflammatory pathway, though, associated with inhibiting NF-κB nuclear translocation, through the upregulation of IκBa, neurotrophin release, and likely stimulation of autophagy 72,95. Consequently, LIPUS, likely stimulates endogenous anti-inflammatory pathways through similar signalling pathways.

### 3.3 CD200/CD200R1 signalling

In peripheral tissues, LIPUS has been used to accelerate both wound and fracture healing 7,97. Investigating the effect of LIPUS on bone fracture healing in pre-osteoblasts, both the upregulation of bone morphology genes and CD200 mRNA was observed 76. While implicated as a possible modulator of bone mass in fracture healing, the CD200/CD200R axis has been proposed as a therapeutic treatment for neuroinflammation, indicating increased CD200 as a possible LIPUS induced anti-inflammatory pathway. CD200 is an inhibitory transmembrane glycoprotein from the immunoglobulin domain super family, ubiquitously expressed across immune cells; signalling through its receptor, CD200R1, leads to the suppression of the inflammatory response 98. Intracellularly, CD200/CD200R1 signalling is propagated through a docking protein 2 (Dok2) and Ras GTPase activating protein (RasGAP)-mediated pathway 98, which leads to the suppression of the phosphorylation of Akt, and extracellular signal-regulated kinase 1 and 2 (ERK1/2), which is induced in inflammation and associated with pro-inflammatory cytokine production 99. Due to its anti-inflammatory function, and dysregulation in inflammation 40,100-103, targeting CD200 has been proposed as a potential treatment for neurodegenerative diseases. Through the administration of either blocking or agonistic antibodies, the CD200/CD200R1 pathway has been observed to promote inflammation resolution 34,100,103; however, the determination of CD200 function was predicated on intervention prior to the induction of, or during an acute inflammatory state. As such, immune cells, especially microglia, were primed with no suppressive mechanism in the case of inhibitory antibody administration, or their inflammatory function was suppressed over that of the homeostatic state, with CD200R1 agonist administration. A recent study discovered that the inflammatory state of the immune cells suppressed by CD200/CD200R1 signalling is critical to this pathway's function when activated in these cells - peripheral blood mononuclear cells (PBMCs) pre-treated with IFNα exhibited altered CD200/CD200R1 signalling compared to untreated PBMCs, either losing the suppressive effect on PBMC activation, or potentiating the inflammatory response 99. Reactive, pro-inflammatory microglia require intracellular caspase-3 activation to mount an immune response, and its blockade prevents microglial activation in response to inflammatory stimuli 105. As mentioned above, immunosuppressive CD200/CD200R1 signalling requires both Dok2 and RasGAP to inhibit both Akt and ERK1/2 activation, and exert its anti-inflammatory effect; with caspase-3 activation, RasGAP is cleaved, which prevents binding to Dok2 resulting in the differential suppression of the Akt pathway, while ERK1/2 and downstream mTOR activation are unaffected 99. Consequently, CD200/CD200R1 loses its homeostatic function in inflammation, a notion often overlooked when investigating the pathway prior to an inflammatory stimulus.

Several further studies looked into the function of CD200/CD200R1 signalling in the context of neuroinflammation, in models of EAE 30, chronic neuropathic pain 106, and aging 102. Subcutaneous injection of 100 μg of CD200Fc, a recombinant CD200 protein, was conducted every other day following disease onset in EAE, to the conclusion of the study, or from day 20-30 to target the chronic phase 30. Administration of CD200Fc at disease onset significantly reduced EAE severity, microglial activation, and prevented macrophage infiltration, as measured by the microglial/macrophage marker CD11b - T cells were largely unaffected. Additionally, pro-inflammatory cytokine levels in CNS and splenic tissue were reduced with CD200Fc administration, while *in vitro* experiments demonstrated a dose dependent effect, with functional benefits observed at lower concentrations in microglia, and higher concentrations 30 in astrocytes. While T cell behaviour was not affected, microglia and dendritic cells isolated from CD200Fc treated mice, in the chronic stage of EAE, demonstrated impaired antigen presenting ability through both reduced MHCII expression, and an inability to stimulate naïve T cells in an *in vitro* assay. The beneficial effect of CD200Fc administration in EAE potentially acts through the reduced activation of T cells upon CNS infiltration 30, which is supported by a reduction in IL-2 production; however, CD200Fc did not alter the expression of markers of activated CD11b+ cells, such as CD86 and CD40, indicating that, in chronic inflammation, CD200Fc affects the adaptive immune response without altering CD11b+ cell reactivity. Flow cytometric analysis at the chronic stage of EAE revealed that CD200Fc administration induced CD11b+ cell apoptosis, based on Annexin V staining, while sparing oligodendrocytes and astrocytes. While caspase-3 activation was also increased in CD11b+ cells with CD200Fc administration 30 this was not used as a marker for apoptosis, as in microglia caspase-3 is necessary for, and associated with, a pro-inflammatory, reactive phenotype 105. As observed in the EAE model, CD200Fc administration in chronic inflammation is associated with the depletion of the local reactive microglial population, and inhibition of antigen presentation in acute stages of inflammation, contributing to reduced disease severity. Classically, the CD200/CD200R1 interaction is associated with the inhibition of the ERK1/2 and Akt signalling pathways, suppressing a pro-inflammatory phenotype. However, in chronic inflammation, CD200/CD200R1 signalling can become rewired through the cleavage of RasGAP by caspase-3 99, leading to selective suppression of Akt, propagation of ERK1/2, and concomitantly increased caspase-3 activation. Low caspase-3 activity is associated with RasGAP cleavage at position 455, generating an apoptotic C-terminal fragment, and an anti-apoptotic N-terminal fragment; high caspase-3 activity results in the further cleavage of the N-terminal fragment, resulting in C-terminal-induced apoptosis 107, constituting a potential pathway behind the observed CD200Fc induced apoptosis of reactive CD11b+ cells. With CD200Fc treatment, caspase-3 activation was increased in CD11b+ cells with CD200Fc treatment, while this was not observed in oligodendrocytes or astrocytes, indicating a potentially rewired signalling pathway in microglia. While apoptosis of reactive microglia is beneficial in reducing the inflammatory response, it should be noted that microglia can promote remyelination as well 22, implying an inherent limitation of CD200Fc treatment in eliminating reactive microglia, rather than altering cell phenotype 108.

Similarly, in a chronic constriction injury model of neuropathic pain, CD200Fc administration resulted in the reduced expression of pro-inflammatory cytokine mRNA, as well as reduced CD11b protein in the spinal cord 106, although it is unclear whether this observation is derived from CD11b+ cell apoptosis, or decreased cellular infiltration. Curiously, both CD200 and CD200R1 expression is upregulated in the neuropathic pain model, and restored to homeostatic levels following the intrathecal administration of 10 μg of CD200Fc 106, potentially indicating the inability of basal levels of CD200/CD200R1 signalling to ameliorate inflammation. In naïve aged (20-22 month old) male mice, intrahippocampal injection of 10 μg of CD200Fc induced a reduction in MHC II expression on CD11b+ cells in the hippocampus, suggesting reduced capability of antigen presentation, but did not affect CD11b or CD68 expression, markers of a pro-inflammatory phenotype 102. However, CD200Fc treatment reduced IFNγ-inducible protein 10 (IP-10) and monocyte chemoattractant protein-1 (MCP-1) mRNA expression, chemokines associated with monocyte and lymphocyte recruitment, in hippocampal tissue. As T cell infiltration into the CNS occurs in aged mice, the reduction in inflammation is likely derived from impaired antigen presentation, and T cell reactivation upon CNS infiltration; while lymphocyte infiltration was not investigated directly, suppressed IP-10 production following CD200Fc treatment may further indicate decreased T cell infiltration 102. CD200Fc administration during the course of inflammation differentially affects activated and quiescent immune cells, inducing apoptosis in reactive CD11b+ cells, and reducing their antigen presentation capacity, while suppressing immune activation in quiescent cells - as such CD200Fc may preferentially affect T cell driven inflammation. Furthermore, CD200R1 agonists may act in a dose dependent fashion, as *in vitro* treatment with 3 μg/ml of OX108, a CD200R1 agonistic antibody, to a cell culture of reactive PBMCs resulted in a loss of immunosuppressive CD200/CD200R1 signalling, instead propagating inflammatory signalling compared to controls, whereas higher doses of CD200Fc were associated with reduced pro-inflammatory cytokine production in microglia (10 μg/ml) and astrocytes (100 μg/ml) respectively, accompanied by decreased disease severity 30,102,106. This suggests that the effect of CD200R1 agonists on downstream signalling pathways may be dependent on the strength of receptor activation. The overexpression of CD200, and treatment with CD200R1 agonists, results in increased production of GDNF 104 and the mitigation of inflammation 30,102,106, which may belie the function of endogenous CD200/CD200R1 signalling in activated immune cells 99. CD200 also plays a role in sex differences in the immune response, with its loss accentuating the difference between male and female TLR7 mediated IFNα production 109, and the induction of reactive microgliosis in the absence of an immunological challenge 34. This is mirrored by an accompanying, regionally specific increase in basal neuroinflammation in aged female mice, as well as increased caspase-3 activity following stroke 110, compared to aged male mice 111,112 - consequently, loss of homeostatic CD200/CD200R1 function may exacerbate sex-differences in inflammation, and propagate DAMP mediated pro-inflammatory feedback loops in females, via TLR7.

## 4. Ultrasound and inflammation - hyperthermic mechanisms of resolution

In the context of wound healing, LIPUS has been beneficially associated with the induction of HSP70 in gingival tissue following mucoperiosteal flap surgery, as well as increased bone regeneration 97. The HSR is a phylogenetically conserved, stress-induced protective mechanism activated in response to hyperthermia, oxidative stress, and shear stress, among others 8. Currently, the heat shock response is not often considered in the mechanistic mitigation of chronic inflammation observed following LIPUS treatment, due to the expected insignificant increases in the temperature of target tissue 7. However, LIPUS has been observed to induce the upregulation of HSP90 64 and HSP70 97, both of which were correlated with beneficial downstream effects, including increased CBF and reduced inflammation. Murine physiology dictates that an increase in CBF will concomitantly increase brain temperature, due to the reduced surface area to volume ratio of the brain, retaining, as opposed to diffusing, heat 113; however, the LIPUS mediated CBF increase is unlikely to induce the HSR alone. Applying laminar fluid stress of 15 dynes/cm^2^ (1.6 Pa) to an *in vitro* culture of endothelial cells resulted in an increased association between HSP90, and eNOS, without increasing the expression of HSP90 114. The peak rarefactional pressure in low intensity ultrasound is on the order of mega-pascals 115, necessitating the consideration of both altered intracellular HSP behaviour and affinity, as well as increased expression following LIPUS treatment 64,97.

As a commonly activated protective pathway, across mechanical or thermal stimulation, the endogenous activation of the HSR requires further investigation as a treatment for chronic inflammation. While LIPUS is the dominant ultrasound modality under investigation when considering treating inflammatory diseases, ultrasound-mediated hyperthermia constitutes an underexplored treatment modality. Hyperthermia is a state where tissue is elevated above resting biophysical levels; presently, the most common use of therapeutic ultrasound, to focally increase tissue temperature, is in the context of HIFU, where temperatures can exceed 60 ^o^C 11,13 in thermal ablation, and reach acceptable peak values of 40-45 ^o^C 11 in mechanical ablation. The febrile range constitutes a physiologically tolerable increase in body temperature, often in response to infection in the form of a fever, to stimulate a series of heat sensitive signalling pathways - at the ultrasound focus in HIFU, these temperatures far exceed the febrile range, reaching lesional potential for use in functional neurosurgery 5 or tumour ablation 6. However, these targets may overlap with comorbid inflammation 13, as inflammation is a risk factor for tumour development, and tumours themselves can generate a local, persistent inflammatory response associated with growth 116; margin effects of ultrasound-mediated hyperthermia, in the febrile range, may affect surrounding inflammatory cells. In the direct application of hyperthermia to pathological inflammation, tissue health must be minimally affected, requiring an alternate treatment modality to HIFU. Ultrasound-mediated mild hyperthermia uses the same principles of high intensity transcranial MRgFUS, while instead limiting temperature increases to physiologically tolerable levels in the febrile range, thereby enabling functional application in neurodegenerative or inflammatory disease. The intersection of febrile range temperatures in inflamed tissue, by deliberate targeting, or edge, effects, necessitates the comprehension of the mechanisms and impact of hyperthermia on chronic inflammation. Additionally, ultrasound-mediated hyperthermia is compatible with real time imaging using MR thermometry, allowing for precise, localized targeting and treatment monitoring in the febrile range.

### 4.1 Low intensity pulsed ultrasound as a regulator of the heat shock response

Fever is a highly conserved physiological mechanism associated with an enhanced response to infection, acting as an adjuvant to the inflammatory response in acute temporal proximity to an immune challenge 8,117 inducing increased pro-inflammatory cytokine release 118; however, extreme fever-related inflammation is deleterious. Within the febrile response, hyperthermia is often delayed from the activation of the innate immune system, and it has been proposed that this enables the endogenous regulation of the immune response, acting as a shutdown mechanism to prevent unregulated inflammation and tissue damage 118. Accompanying the febrile response is an increase in core temperature of 1-4 ^o^C, which activates the HSR. Heat shock factor 1 (HSF1) is a transcriptional factor which propagates and initiates downstream HSR signalling 8, and is bound to HSP90 in the cytoplasm, maintaining a quiescent state 119. Upon stimulation with hyperthermic temperatures, HSP90 and HSF1 dissociate 120, and HSF1 trimerizes and translocates to the nucleus, facilitating the transcription of HSR-related genes 119 and HSPs, notably HSP90, HSP70, HSP60, and HSP27 8. HSF1 activation occurs over a dynamic thermal range, coincident with temperature and durational increases, resulting in the proliferation of HSP production 8; temperatures observed in HIFU, well above the febrile range, denature HSPs, thereby mitigating their efficacy and functional propagation 11. Intracellular HSPs execute a chaperone function, binding perturbed proteins for refolding, degradation, or sequestration from the surrounding intracellular environment, protecting the cell from the toxicity induced by dysregulated proteins 8. Currently, there is a wide array of heterogeneous evidence as to the impact of exogenous, or extracellular HSPs, with conflicting reports of pro- or anti-inflammatory functions, although this is likely a function of cell activation state 121 and release mechanism 122.

As hyperthermia has been found to be detrimental in disease etiology and inflammation in models of acute CNS insult, such as mild traumatic brain injury 123, it has largely been underexplored as a therapeutic strategy for chronic inflammation, despite promising results in peripheral tissues 124. Aside from the upregulation of HSPs, HSF1 directly modulates the release of pro-inflammatory cytokines 53,121,125,126 and promotes neurotrophic factor release 127. Elevated temperatures have been associated with the reduction of pro-inflammatory cytokines during fever 8, indicating a mechanism of endogenous regulation of inflammation instigated by the HSR. Heat shock related gene expression, or transcription, is mediated by the heat shock element (HSE), a transcriptional regulatory element in the promoter of genes modulated in a stress response 126. Notably, IL-1β 128, TNFα 129, and BDNF 127 all express HSE sites, enabling modulation of their transcription by HSF1. For IL-1β and TNFα, HSF1 acts as an inhibitory factor, preventing cytokine transcription by binding to the HSE 128,129, thereby inhibiting the inflammatory response; this has been show to result in a marked reduction in the levels of pro-inflammatory cytokines produced by human monocytes in response to LPS stimulation, and can be induced both by hyperthermia and exogenous HSF1 treatment. While acting as an inhibitor for IL-1β and TNFα, HSF1 interaction with the BDNF HSE binding site resulted in the upregulation of BDNF in the hippocampus, and a neuronal cell culture 127. When detailing the upregulation of BDNF in microglia in response to LIPUS, the mechanism of action was not investigated outside of the BDNF/TrkB positive feedback loop 81. Consequently, the *in vivo* mechanism of BDNF upregulation with ultrasound treatment may be mediated by HSF1 and the HSR.

Downstream of HSF1, HSPs perform several neuroprotective functions which may aid in the resolution of chronic inflammation. HSP70 is a protein of particular interest due to its specific upregulation through HSF1 and its implications in numerous neurological diseases. Thought of as neuroprotective when acting intracellularly, the extracellular role of HSP70 is contentious, with evidence of both protective 126 and inflammatory functions 122. HSP70 is capable of acting as an endogenous ligand for TLR4 126 and TLR7 130, through direct, or EV, release; however, the induced signalling pathways are not identical to those mediated by PAMPs. Upon HSP70-mediated TLR7 activation, a cell-membrane bound, rapid, transcription-independent signalling pathway is induced, which upregulates phagocytic activity via phosphatidylinositol-3-kinase (PI3K), an upstream Akt signalling molecule 82, and p38 mitogen-activated protein kinase (MAPK), a generally pro-inflammatory although context-dependent molecule 131 130. As such, while HSP70-TLR7 interaction increased the relative expression of TNFα compared to homeostasis in unstimulated macrophage cells 130, it is unclear whether a pro- or anti-inflammatory effect is induced, or how the signalling pathways will change in chronic inflammation, although increased phagocytosis is thought to be beneficial in neurodegenerative diseases 96,104.

Interpreting the consequences of HSP70 interactions with TLR7 necessitates additional context for the role of PRRs, as the signalling response bifurcates according to the activating ligand. The PI3K-Akt pathway is an essential component of both eNOS release and phosphorylation 132, which acts as a potent anti-inflammatory signal. In whole brain LIPUS treatment of a vascular dementia model, the blockade of eNOS was sufficient to abrogate the anti-inflammatory effect of LIPUS in mice 64. Phosphorylated Akt is essential for eNOS activation, and acts as a downstream target for both the inducible and constitutive forms of HSP70; blockade of constitutive HSP70 with small interfering RNA resulted in a decrease in PI3K production in human umbilical vein endothelial cells in response to vascular endothelial growth factor (VEGF), while the knockdown of the inducible form reduced Akt phosphorylation independent of PI3K activity 132. In both cases, the activation of eNOS was eliminated. Traditionally, the connection between HSPs and eNOS has canonically been linked to HSP90 114, which associates with phosphorylated Akt, thereby preventing its inactivation, and allowing downstream eNOS activation. However, the requirement of HSP70 expression for eNOS activation in both *in vitro* models of VEGF administration, and *in vivo* mouse models of hind limb ischemia 132, potentially indicates a larger integration with the HSR. Cellular stress, and the HSR, has been shown to alter protein function at homeostasis 114, independent of transcriptional regulation. HSP70 affinity and release may be altered during periods of cellular stress, resulting in increased interactions between constitutive HSP70 and TLR7, and the subsequent activation of PI3K 130. Similarly, inducible HSP70 may be necessary as a co-chaperone for HSP90 133 in maintaining Akt phosphorylation during cellular stress. However, while the HSR and HSPs are necessary for the induction and facilitation of many anti-inflammatory signalling pathways, the functional ability to induce gene transcription in response to stress varies with age.

Aging and chronic inflammation are associated with cellular senescence, and the subsequent impaired ability to induce protective signalling pathways, such as the HSR, in response to stress signals. A key protein downregulated with aging and associated with cellular senescence, is the deacetylase sirtuin-1 (Sirt1) 134; the role of Sirt1 as a transcriptional regulator is of interest in the context of chronic inflammation. Cellular senescence is characterized by an inability to respond to the environment and signalling molecules, limiting the ability to transcriptionally respond to external stimuli, and, in chronic inflammation, commonly resulting in a persistent pro-inflammatory phenotype 135. In homeostasis, abundant Sirt1 acts as a transcriptional regulator, interacting with HSF1 and increasing its binding stability to the HSE of target genes by preventing its acetylation, allowing for rapid and sufficient induction of the HSR - in aging, reduced levels of Sirt1 concomitantly increase HSF1 acetylation, impairing the HSR, the subsequent induction of HSPs, phenotypic plasticity, and stress tolerance as a result 136. While aging reduces HSP production regardless of sex 137, females are preferentially affected 138. Estrogen signalling is reduced with age in females, and directly corresponds to eNOS production 66; Sirt1 and phosphorylated eNOS act in a positive feedback loop, with eNOS activating Sirt1, and vice-versa 139, potentially explaining the preferential HSP decline in females as a function of estrogen signalling. Contextualizing Sirt1 as a component of the HSR necessitates the consideration of potential non-canonical HSP70 interactions, with TLR7, and in forming a stabilizing complex with HSP90 and phosphorylated Akt. Consequently, while previous studies have identified eNOS as the main regulator of the anti-inflammatory effect of ultrasound 64, a wider context emphasizes an overarching signalling pathway stemming from the HSR acting in a protective, and self-propagating fashion. While eNOS may be necessary, in a model of vascular dementia, the constituent signalling components (HSP70, PI3K, Akt, Sirt1) are also essential; the resultant effect on HSF-1 transcription, and upregulation of HSP70 indicates a closed loop starting with the foundational, canonical components of the HSR, which may be able to reduce cellular senescence, and ameliorate sex differences in chronic inflammation, and aging.

### 4.2 Heat shock response

A recurring theme in the immune system is the context dependent outcome of signalling pathways or stimuli; this carries over to the interaction between HSP70 and TLR4. Treatment of unstimulated macrophages with EV-HSP70 or exogenous HSP70 resulted in an increase in the release of pro-inflammatory cytokines, and NF-κB translocation, and acted as an adjuvant in the inflammatory response to a mycobacterial infection 122. In this context, HSP70 contributes to a more aggressive inflammatory response of infected cells, improving pathogen clearance, and likely functions as a DAMP, activating unstimulated cells for phagocytosis. However, other experiments demonstrated HSP70 administration following LPS monocyte stimulation significantly inhibited TNFα production, while HSP70 pre-treatment before LPS stimulation prevented any increase in TNFα expression 126. Consequently, while there are conflicting reports, HSP70 seems to inhibit or reduce inflammation. Mechanistically, HSP70 binds to TLR4 and induces the downstream activation of HSF1, which intracellularly inhibits pro-inflammatory cytokine release, thereby regulating excessive inflammation 126; this is reflected *in vivo*, as HSP70 induction in sepsis, via hyperthermia, is associated with reduced mortality 140.

While often proposed as the main actors in the HSR, studies investigating hyperthermia after ablation of HSF1, and concomitantly HSP70, demonstrated the downregulation of pro-inflammatory cytokines 141, which was absent from non-heat shocked cells with constitutively active overexpression of HSF1 142. The implication is that the beneficial reduction in inflammation due to heat shock is independent of both HSF1 and induced HSP70, contrary to commonly proposed mechanisms of resolution; however, this conclusion might be too simplistic. In models of inflammation and infection, the HSR is activated as a protective mechanism, resulting in the upregulation of HSF1, and subsequent HSP70 transcription 126, the loss of which increases mortality in models of sepsis 140. Exposure to heat shock, or exogenous HSP70 treatment, leads to HSF1 upregulation - potentially sensitized through a hypoxia inducible factor 1α (HIF1α)-mediated interaction 143 - as well as a rapid recruitment of constitutive heat shock element binding factor (CHBF) to the transcriptional complex 144, which is absent following sole stimulation with LPS 126. Both HSF1 and CHBF interact with the HSE on the TNFα promoter, thereby inhibiting its transcription. The similar anti-inflammatory function of CHBF provides a potential mechanism for the apparent HSF1-independent downregulation of pro-inflammatory cytokines. Despite this, the protective influence of HSF1 on the HSR is still apparent, but functional benefits may only be discernible under physiologic stressors, as the affinity and cellular behaviour of HSPs can be altered by shear stress, and VEGF 114. This bears emphasis, as prior research has described a HSP70-mediated cessation of NF-κB inhibition, through the stabilization of IKK 145, preventing its insolubilization during hyperthermia 146 - while this would be detrimental in chronic inflammation, the model described returned IKK activity to baseline levels in response to heat shock 145, but not DAMP or PAMP stimulation. NF-κB plays an important role in cell homeostasis independent of inflammatory function as well 147, and HSP70 stabilizes this pathway in response to heat shock, while accomplishing an inverse function in inflammation 148.

Further investigation of the CHBF has revealed identical immunological function to the Ku autoantigen 149, a signalling molecule integral to the repair of double strand DNA (dsDNA) breaks, composed of Ku70/80 subunits. The presence of Ku70/80 in heat shock related pathways has generally been considered a suppressor of the HSR, with overexpression of the Ku70 subunit directly inhibiting HSP70 induction 150, although this could be a non-physiological response to supra-endogenous levels of a specific subunit 150, as Ku70 ablation also inhibits HSP70 expression 151; while seemingly contradictory, Ku70/80 fills a complex regulatory function, facilitating HIF1α- 144 and HSR- 152 induced gene transcription. Gene transcription is regulated by RNA polymerase II (Pol II), which transcribes mRNA precursors from the DNA template - in stimulus induced gene transcription, Pol II is paused after binding, primed to facilitate rapid transcription 153. An activating stimulus, such as hyperthermia or hypoxia, facilitates the agglomeration of a signalling complex capable of releasing the transcriptional brake on Pol II, resulting in the rapid transcription of downstream targets 144,151-153. Ku70/80 are key components in promoting stimulus-induced gene transcription, and are specifically required for HSP70 protein expression 151,152; however, Ku70/80 overexpression to supra-physiological levels can result in transcriptional suppression through outcompeting other transcription factors for binding to DNA 154. Regardless, the reparative function of the Ku autoantigen in dsDNA breaks, a source of inflammation induced by ionizing radiation 18, implies a protective function. As a competitor of HSF1 for HSE binding 126, as well as a facilitator of HSP70 transcription 151,152, the effective increase in CHBF levels following heat shock may result in the preferential targeting of pro-inflammatory cytokines in inflammation, as well as facilitating the HSF1-mediated induction of HSP70, resulting in the dual upregulation of HSF1 and CHBF in response to exogenous HSP70 stimulation 126, while acting as an inducible HSP70 suppressor in homeostatic conditions. Contextually, the HSR is composed of multiple HSE binding factors capable of inhibiting pro-inflammatory cytokine transcription, and is not solely dependent on HSF1; furthermore, there appears to be a distinct difference between thermal activation of the HSR, accompanied by extracellular HSP70 release, and pathogen-induced stress, through the activation of different transcription factors 155. Several functional changes likely occur during the non-pathogen induced activation of the HSR, with variations in the concentration, downstream effect, and release of HSPs, as well as HSE binding factors, constituting an anti-inflammatory response. As such, the HSR and HSPs can holistically be described as homeostatic regulators.

Intracellularly, HSP70 mitigates inflammation downstream of HSF1. The inflammasome is an inflammatory intracellular signalling complex implicated in a number of neurodegenerative diseases 156; upon activation of PRRs by PAMP/DAMPs, NLRP3 transcription is increased 53 - a key component of the well characterized NLRP3 inflammasome. Further endogenous signalling results in the agglomeration of NLRP3, the apoptosis-associated speck-like protein containing a caspase recruitment domain adaptor protein, and pro-caspase-1, resulting in IL-1β activation and the induction of NLRP3 inflammasome activation in surrounding cells 53. Whole body heat shock of 42^ o^C for one hour induced HSP70 overexpression in a mouse model, which was found to bind to NLRP3, inhibiting inflammasome formation and IL-1β production, independent of HSF1 53; of note, the hyperthermic parameters that were used upregulated HSP70, but no other HSPs investigated. In cardiac tissue HSP70 is predominantly induced at higher temperatures 157 while HSP90 has a lower threshold of activation 158, indicating HSP expression is both tissue and temperature specific - this holds within CNS cells 159. A downstream signalling molecule induced by inflammasome activation 160, as well as TNFα signalling 51, is the endogenous TLR4 ligand HMGB1, which demonstrated an inverse relationship in expression to HSP70 in acute pancreatitis, with high HMGB1 and low HSP70 expression being associated with mortality 161; HMGB1 release is abrogated following hyperthermia 125, potentially acting through HSP70/HSF1-mediated inflammasome and TNFα inhibition. Hyperthermia has further been shown to induce nuclear factor erythroid 2-related factor 2 (NRF2) 162, downstream of immune-responsive gene 1 (IRG1) and itaconate 163, a factor associated with reduced caspase-3 activation and neuroinflammation in a model of spinal cord injury 163. NRF2 is a transcription factor capable of cross talk with HSF1 164, and IRG1 and itaconate are both potent inhibitors of IL-1β and NLRP3 inflammasome activation 163. While hyperthermia has to date only been associated with increased NRF2 in this pathway, high intensity ultrasound (HUS) has been demonstrated to induce IRG1 and itaconate production as well 165, potentially indicating a common signalling pathway responsible for NRF2 upregulation.

NRF2, aside from its association with the IRG1 and itaconate pathway, is capable of accelerating and changing the dynamics of the potent anti-inflammatory feedback regulator TNFα-induced protein 3 (TNFAIP3/A20). TNFAIP3 is a negative feedback regulator of NF-κB signalling, which contributes to the dynamic, oscillatory expression pattern of NF-κB, inhibiting IKK activation and downstream NF-κB nuclear translocation 166. While often characterized in binary terms, NF-κB activation is modulated by a complex series of signalling pathways, and underlying self-regulated inhibitory molecules, which induce a damped, periodic behaviour in NF-κB nuclear translocation at both the single cell 167,168, and population level 169. Conceptually, the dynamics of NF-κB transcription enables the encoding of gene expression in a context-dependent fashion, facilitating pleiotropic downstream effects. In the context of the HSR, TNFAIP3 has demonstrated temperature sensitive expression in response to a TNFα inflammatory challenge in SK-N-AS neuroblastoma cells, with an earlier upregulation in cells incubated at 40 ^o^C compared to 37^ o^C 168. Increased TNFAIP3 expression was correlated with an early upregulation of a number of NF-κB related genes, associated with both the immune system and the inflammatory response - a difference which was absent at later timepoints 168, indicating a time and state dependent alteration in inflammatory signalling. The acceleration in TNFa-induced TNFAIP3 expression in response to hyperthermia could be mediated by the upregulation of NRF2 downstream of the HSR. The TNFAIP3 promoter contains an antioxidant response element (ARE); NRF2 has been shown to bind to TNFAIP3's ARE, and subsequently upregulate its expression in bone marrow derived macrophages 170. As an essential regulator of inflammation, perturbations in TNFAIP3 are associated with a number of autoimmune diseases 166, and its absence leads to mortality 171. Consequently, the upregulation of TNFAIP3 has a potentially beneficial effect in treating inflammation and is downstream of pathways induced by the HSR. While attempts have been made to model the cross-talk between the HSR and NF-κB signalling pathways 167, largely predicated on the presumed inhibitory functions of HSFs and HSPs, it remains to be determined whether the assumptions and simplifications made hold true in chronic inflammation. Regardless, it is evident that the dynamics of signalling pathways are more complex than generally considered and are functionally capable of different downstream effects in response to the same stimulus; consequently, this may offer a partial explanation for the ability of the HSR to act as both an inflammation-promoting, and resolving mechanism depending on the cellular context.

Emphasizing the time and context dependent effect of hyperthermia, is the exacerbation of neural injury when hyperthermia is applied immediately following mild traumatic brain injury (TBI) in a murine model 123 - LIPUS, in contrast, has been demonstrated to reduce edema and improve therapeutic outcomes in both an acute and longitudinal timeframe 80. Recently, EVs have received increased interest due to their implication as both a mechanism of disease and resolution, and may provide insight into the mechanistic immunological differences between LIPUS and hyperthermia - EV release is increased by both LIPUS 172 and hyperthermia 173, while EV contents are differentially modulated. FUS hyperthermia treatment of *in vitro* glioma cells induced a proteomic shift in EV contents, inducing a beneficial, pro-inflammatory, anti-cancer profile 173, similar to the immune adjuvant effect of acutely applied hyperthermia in a model of peripheral LPS infection 117; this was accompanied by a decrease in major vault protein, which has been associated with sorting miRNAs for EV loading 174. Alternately, bone marrow derived dendritic cells exposed to LIPUS exhibited an anti-inflammatory shift in EV miRNA contents 175, and subsequently reduced inflammation. Consequently, the context dependent effect of the HSR may manifest as changes in EV loading machinery, in addition to changing the dynamics and timing of certain immune signalling pathways. While there have been conflicting results on the pro- or anti-inflammatory effect of HSP70, whether exogenous or from EVs, these may be localized to the method of isolation, timing, and cellular origin or context when administered, as EV contents will impact downstream signalling. Regardless, the anti-inflammatory effect, and potential of HSPs is well established.

Beyond inhibiting NLRP3 inflammasome formation, HSP70 has a more fundamental effect on immune cell reactivity. Microglia require caspase-3 signalling for pro-inflammatory cytokine release 105, while caspase-9/caspase-3 activation induces apoptosis in neuronal cells 176. Caspase-9, activated via the apoptotic protease-activating factor 1 (Apaf-1), is an endogenous activator of caspase-3, cleaving the pro-caspase form and subsequently inducing apoptosis 177. Overexpression of HSP70 does not affect pro-caspase-9 transcription, but instead prevents its cytosolic activation, likely through interactions with Apaf-1, thereby inhibiting both caspase-9 and downstream capsase-3 signalling 9. Apaf-1-mediated caspase activation is a constituent of the intrinsic apoptosis pathway, while caspase-mediated microglial reactivity is dependent on the extrinsic pathway, which is characterized by upstream activation of caspase-8 leading to pro-caspase-3 cleavage 105; in a model of ischemic stroke, transgenic HSP70-overexpressing mice were found to have decreased caspase-8 activation 178, indicating the ability of HSP70 to inhibit both intrinsic and extrinsic pathways of apoptosis. Notably, the apoptotic pathways exhibit sex-driven differences in activation, with female mice exhibiting significantly more caspase-8 and capsase-3 activity following ischemic stroke, and reduced neurological deficits after administration of a pan-caspase inhibitor 110; this effect was not observed in male mice. Additionally, in female rats, the ability to upregulate HSP70, and HSP27 in the brain following heat shock is reduced compared to their male counterparts 138, indicating a potentially reduced ability to abrogate caspase-3 mediated inflammation - this is consequential when considering that caspase-3 is implicated in CD200/CD200R1 dysregulation 99, a pathway which at homeostasis mitigates female biased sex-differences 109 in the immune response. Regardless, HSP70 is implicated in preventing neuronal apoptosis 9, and microglial activation, while potentially acting as a rectifier of immune-based sex differences.

Downstream of caspase signalling, hyperthermia-induced HSP70 in astrocytes exposed to oxygen-glucose deprivation interacts with JNK and p38, inhibiting their phosphorylation, subsequent nuclear translocation, and downstream inflammatory cytokine production - further, HSP70 prevents IκB phosphorylation, resulting in the continued sequestration of NF-κB in the cytoplasm 148. Transcriptional changes confluent with hyperthermia included a reduction in MMP-9 mRNA expression 148, demonstrating a potential pathway leading to increased BBB stability, although hyperthermia is associated with increased acute BBB permeability 179. The combination of both aging and inflammation can result in lymphocyte infiltration in the CNS, and is associated with detrimental outcomes; interestingly, HSPs can act as antigens for T cells, and modulate their response 180. As HSPs are phylogenetically conserved, from bacteria to mammals, there is a self-reactivity of T cells to endogenous HSPs, likely mediated through cross-recognition of T cells sensitized to bacterial HSPs 180. In disease, HSP-reactive T cells exhibit an immunoregulatory phenotype, and contribute to inflammation resolution 180. Stressed cells induce HSPs, which can be processed and presented as antigens via MHC I/II on APCs 180, which act as a signal of cellular distress. Induced in the later stages of inflammation, or infection, self-HSP-reactive T cells act in an ameliorating fashion, recognizing cells stressed by excessive inflammation, and correspondingly secreting anti-inflammatory cytokines to promote resolution 181. Hyperthermia could act as an inflammatory shutdown mechanism, through the adaptive immune response, by the induction of the HSR, and subsequently this regulatory T cell response.

In disease, HSP70 and HSP90 are two of the more studied HSPs, although there remains interest across other HSP family members, potentially displaying anti-inflammatory function. Of note, HSP27 is a small intracellular HSP which has been shown to improve cognitive function in mouse models of AD, without altering caspase-3 activation 182, and is thought to inhibit JNK phosphorylation 183 - regardless of the mechanism, HSP27 is thought to be protective. Similarly, HSP60 has been shown to induce regulatory T cells, and mitigate chronic inflammatory disease pathology 184,185, and can further act as a ligand for the TREM2 receptor 185, which induces a phagocytic, anti-inflammatory phenotype in myeloid cells. Induction of HSP release is a constitutive process, and is endogenously activated in the febrile range; ultrasound-mediated hyperthermia has been demonstrated to induce HSP transcription at lower temperatures than solely temperature-based methods 186. For example, a similar adjuvant effect was observed with electric stimulation (ES)-mediated hyperthermia, compared to ES alone, in a model of chronic prostatitis 187.

**Figure 3:**
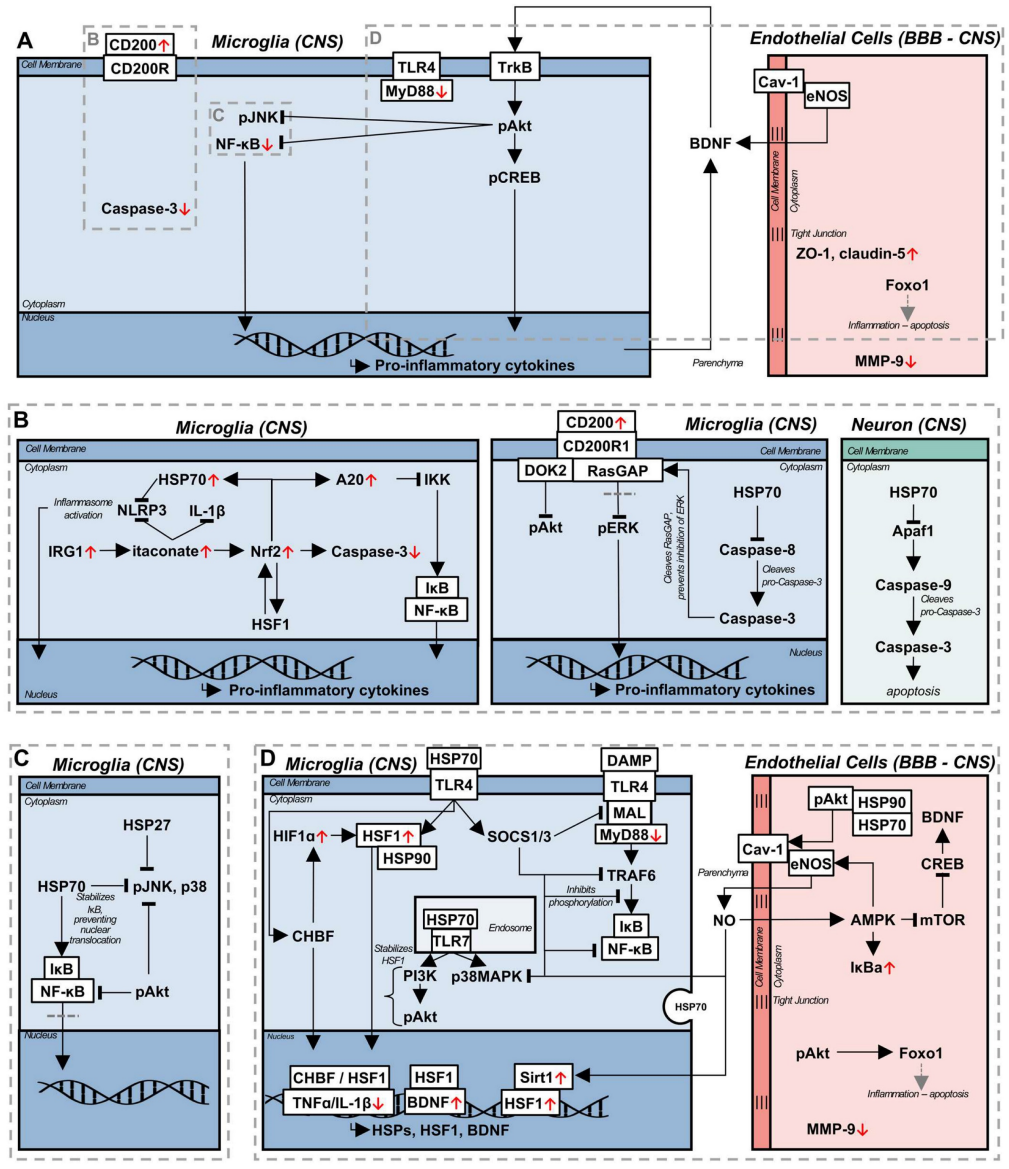
Comparison of the observed anti-inflammatory effects of LIPUS, with proposed HSR induced anti-inflammatory signalling pathways as an explanatory mechanism. *(A)* Experimentally observed changes in key signalling pathways proposed as the mechanisms behind LIPUS mediated resolution of inflammation. *(B)* Signalling pathways upregulated during the HSR, and mild hyperthermia, capable of inhibiting inflammasome formation, and downregulating caspase-3 activation (left). Under inflammatory conditions, CD200/CD200R signalling can become dysregulated, with caspase-3 cleaving RasGAP, leading to the differential inhibition of pAkt, while losing the ability to inhibit ERK activation. Microglial activation is dependent on the caspase-8/caspase-3 pathway, allowing HSP70 to both inhibit activation, and restore homeostatic CD200/CD200R signalling (middle). HSP70 also inhibits the neuronal apoptosis pathway (right). *(C)* In addition to the proposed LIPUS mediated inhibition of JNK phosphorylation, and NF-kB activation, through pAkt, HSP27 and HSP70 inhibit pJNK, while HSP70 additionally prevents p38 MAPK phosphorylation, and IKK mediated degradation of IkB. *(D)* HSP70 has been described to bind to TLR4, upregulating SOCS1/3 which directly inhibits MAL and subsequent MyD88 TLR4 signalling complex formation, and pro-inflammatory cytokine production. Additionally, HSP70 and TLR4 interactions are associated with increased HSF1, and CHBF transcription, both of which are capable of binding to the heat shock elements of target genes, downregulating pro-inflammatory cytokines, while upregulating neurotrophic factors. Crosstalk between stress response pathways, such as the hypoxia and heat stress responses, generates a compensatory, symbiotic cascade of anti-inflammatory signalling pathways, with CHBF, HIF1a, and HSF1 facilitating mutual gene expression. TLR7 and HSP70 have been shown to interact, and activate the PI3k/pAkt pathway, which is implicated in stabilizing HSF1 and upregulating the HSR. Through extracellular vesicles, and direct protein secretion, adjacent cells can communicate and influence the cellular environment, and activation states. In studies of LIPUS, eNOS was characterized as an essential component for an anti-inflammatory effect, and was correlated with the upregulation of BDNF, while a mechanism was not proposed. Pharmaceutical upregulation of eNOS is predicated on a complex of phosphorylated Akt, and HSP90, with potential co-chaperone behaviour of HSP70, eventually leading to NO release. NO stimulates the AMPK/mTOR/CREB/BDNF pathway, potentially explaining the observed relationship between eNOS and BDNF release. Simultaneously, NO upregulates Sirt1, a transcriptional regulator necessary for sufficient induction of the HSR.

Likewise, additional downstream targets of the HSR are implicated in its anti-inflammatory effects. Hyperthermia-mediated resolution of inflammation in a model of chronic prostatitis was not only accompanied by the upregulation of HSP70, but also an increase in suppressor of cytokine signalling 3 (SOCS3) expression 187. The SOCS protein family are potent inhibitors of pro-inflammatory cytokine feedback signalling, especially following TLR activation 188. Both SOCS1 and SOCS3 are upregulated downstream of the HSR, with HSP70 inducing SOCS1 expression through TLR4 activation 189, targeting TRAF6, MAPK, and the p65 subunit of NF-κB 188, which are implicated in a number of pro-inflammatory pathways. Additionally, the HSR-related upregulation of SOCS1 may explain the previously reported reduction in TLR4 expression 79, and subunit association 190 following ultrasound, through the direct degradation of the MAL adaptor protein 191, essential for MyD88 dependent TLR2/4 signalling. In LIPUS, this mechanism has previously been attributed to mechanical perturbation of the cell membrane, although this has not been verified 190. Preventing degradation of IκB, and subsequent NF-κB nuclear translocation, has also been attributed to phosphorylated SOCS3 activity, which shields the phosphorylation sites of IκB from IKK 192. Consequently, both SOCS1 and SOCS3 act as potent inhibitors of pro-inflammatory cytokine signalling in response to stress and are upregulated as part of the HSR downstream of HSP70. Colloquially, the HSR is often simplified and attributed to the upregulation of HSF1 and its target genes in response to stress, especially HSP70; however, it is clear that the HSR indirectly upregulates a number of signalling pathways (Figure 3), initiating a protective cascade, while modulating intracellular protein function depending on the stressor, and phenotypic state.

## 5. Summary

Investigation into ultrasound as a treatment for chronic neuroinflammation has raised interest in recent years, after demonstrated successes in accelerating healing in wounds, bone fractures, and reducing inflammation in peripheral tissues 7. Promising results peripherally have been followed by the application of LIPUS in CNS models of chronic inflammation and neurodegenerative disease with similarly beneficial results, albeit using heterogeneous ultrasound parameters. The success of LIPUS has been attributed to the upregulation of neurotrophic factors, such as BDNF and GDNF, the upregulation of inhibitory signalling pathways (CD200/CD200R1), increased BBB stability, increased eNOS activation, and the inhibition of pro-inflammatory cytokine release. However, the broad spectrum of described ameliorating pathways established with heterogeneous experimental parameters indicates a potential common upstream pathway mediating these changes.

The effect of hyperthermia has been largely disregarded in the translation of thermal therapy from peripheral application to the CNS. While mild hyperthermia has an agonistic effect on the immune response in acute inflammation, it appears to act as a regulatory factor in chronic inflammation, mitigating the release of pro-inflammatory cytokines and promoting resolution. The HSR constitutes the upstream mechanism of this effect, inducing a protective, anti-inflammatory environment in response to stress, upregulating HSF1, and HSPs. Evaluation of common signalling pathways between hyperthermia and LIPUS demonstrate considerable overlap, with the HSR implicated in the upregulation of neurotrophic factors, BBB stability, inhibition of pro-inflammatory cytokine release, and inflammasome disruption. Furthermore, in addition to the upregulation of eNOS production, the HSR has the capacity to rescue inhibitory CD200/CD200R1 signalling, integral in mediating sex differences in inflammation, from a chronic inflammation induced change of function to potentiating the inflammatory response. With this contextual information, the observation of HSP upregulation in models of both wound healing 97 and Alzheimer's disease 64 following LIPUS treatment indicates a potentially confluent integration of the HSR in LIPUS-mediated inflammation resolution - as HSP family and function are mediated by the amount of cellular stress induced, seeming discrepancies in downstream outcomes are easily reconciled.

As HSPs are implicated in immune regulation, the prolonged nature of chronic inflammation potentially indicates a dysregulated HSR, and an upregulation of HSPs incapable of ameliorating the inflammatory response. In sterile inflammation, HSPs may act as immunoregulatory factors via both the innate immune system, through disruption of pro-inflammatory cytokine feedback loops and inflammasome activation, and the adaptive immune response, through the induction of regulatory T cells - if the upregulation of the HSR is impaired or disrupted, inflammation may become chronic. Endogenous stimulation of the HSR thereby constitutes a promising inlet to a cascade of beneficial downstream therapeutic pathways in chronic inflammation, while avoiding potential undesirable side effects of pharmacologically inducing or inhibiting individual signalling molecules.

In describing the potential of the HSR as a common regulator of the beneficial immunological response to both LIPUS and hyperthermia, the mechanisms behind the contextual effect of hyperthermia have not been explored. While there is considerable homology between LIPUS and hyperthermia, only hyperthermia has been recorded as capable of agonizing the immune response in acute inflammation, or infection - to date, there is a paucity of data attempting to explain this phenomenon. While changes in signalling dynamics have been reported in response to hyperthermia, and require further investigation, the immunological context is also influenced by secreted EVs. As a proposed biomarker of disease, and a potential therapeutic target, EV contents give a snapshot of the cellular environment - interestingly, their contents and loading complexes are influenced by both LIPUS, and hyperthermia. While acknowledging the gaps in the literature, hyperthermia and LIPUS may differentially affect EV loading complexes, and EV contents as a result; this may explain an aspect of the temporal plasticity of the immune response to hyperthermia and should constitute a future avenue of investigation.

Similarly, tissue oxygenation levels constitute another area of future research, as oxygenation can be affected by inflammation 193, hyperthermia 194, and LIPUS 64. Contextually, hypoxia can form a positive feedback loop with inflammation, through the production of reactive oxygen species and DAMPs from chronic, sustained damage 193 - cellular survival is dependent on the mutualistic activation of both the hypoxia, and heat shock, response pathways. HIF1α is the primary regulator of the hypoxia response 195, responsible for altering cellular metabolism, oxygen consumption, and is essential in upregulating HSF1 and downstream HSPs during both hypoxia 195, and hyperthermia 143. HIF1α is abundant at homeostasis, but normoxic conditions leads to its degradation, which can be abrogated by the induction of HSP90 196, potentially explaining its upregulation following LIPUS 197, and hyperthermia. Holistically, the HSR and the hypoxia response are linked in a bidirectional modulatory relationship, demonstrated in their activity following heat acclimation in humans 198, and co-interactions. Additionally, hyperthermia and LIPUS are potentially capable of increasing tissue oxygenation, whether through transient, local changes 194, or long term eNOS mediated increase in CBF 64; however, with respect to inflammation, it is unlikely that transient fluctuations in tissue oxygenation exert an immunological effect 193. Regardless, while outside the scope of this review, the bioeffects of hyperthermia and ultrasound - and the cross-regulation of stress response pathways - in the context of oxygenation remain of interest in oncology and require further study in inflammation.

Ultrasound-mediated hyperthermia has been demonstrated to induce greater HSP transcription at lower temperatures than hyperthermia alone, indicating an amplifying effect of the costimulatory thermal and mechanical stress. This is essential in the context of aging, where the HSR, and downstream HSP transcription is compromised 137 - ultrasound is capable of initiating the HSR, and potentially improving transcriptional efficacy lost in aging through the upregulation of the metabolic protein Sirt1 136,199, although the specific mechanisms are unclear. Additionally, ultrasound-mediated febrile range hyperthermia is largely understudied in chronic inflammation, especially with regard to the CNS, despite the inherent benefits associated with the technique. By using both focused ultrasound, and MR thermometry, ultrasound-mediated hyperthermia can be targeted both functionally, and anatomically, while allowing for intra-treatment monitoring. This is important, as some inflammatory and neurodegenerative diseases are localized, requiring the use of spatial as well as functional landmarks for targeting; MRgFUS neurosurgical ablation has resulted in the amelioration of a potentially inflammatory abnormality in the human brain, by an as yet unclear mechanism, necessitating further investigation. Combining both ultrasound and febrile range hyperthermia allows for the induction of the HSR, as well as a concomitant broad spectrum of HSPs and anti-inflammatory pathways, constituting a promising potential therapeutic modality.

## Figures and Tables

**Table 1:** Summary of experiments investigating the biophysical impacts of therapeutic ultrasound, where all effects are compared to a non-US group (i.e. LIPUS compared to SHAM, LPS+LIPUS compared to SHAM+LIPUS). For a more extensive compilation of the effect of LIPUS across cell types, see the review by Xu *et al.* 7.

Model	Experiment	LIPUS Parameters	Biological effect
*in vitro* 76 macrophages: bone marrow derived HUS	measured gene expression 3h post-US LPS: 100ng/mL measured inflammatory response to LPS 3h post-US, 1.5h with LPS (4.5h from start of US)	*f_c_:* 1 MHz *f_R_:* 100 Hz *I_SPTA_:* 3 W/cm^2^ *Duration:* 5 min *Duty cycle:* 20% *Burst length:* N/A *Transducer*: plane	*US:* - apoptosis, ↑Irg1 (mRNA), ↑itaconate (mRNA), ↑Nrf2 (mRNA) *US+LPS:* ↓IL-1b (mRNA), ↓IL-6 (mRNA), ↓Tnfα (mRNA) Verified cell media did not exceed 37 ^o^C
*in vitro* 81 microglia: BV-2 astrocytes: CTX TNA2 LIPUS	LPS: 1 μg/mL LIPUS 12h post-LPS Evaluation at 4, 8, 12, **24 h** post-LIPUS	*f_c_:* 1 MHz *f_R_:* 100 Hz *I_SPTA_:* 10, 20, **30 mW/cm^2^** *Duration:* 15 min (3x5/5 on/off) *Duty cycle:* 50% *Burst length:* 5 ms *Transducer*: plane	Microglia *LIPUS:* ↑BDNF, ↑GDNF, ↑VEGF, ↑p-CREB/CREB, ↑p-Akt/Akt, ↑p-TrkB/TrkB *LPS+LIPUS:* ↑BDNF, ↑GDNF, ↓VEGF, ↓TNFa, ↓IL-1b, ↓IL-6, ↑p-CREB/CREB, ↑p-Akt/Akt, ↑p-TrkB/TrkB, ↓TLR4, ↓MyD88, ↓p-JNK/JNK, ↓p-p65/p65, ↓iNOS TrkB/Akt/CREB/BDNF pathway required for BDNF production Akt inhibits JNK Astrocytes *LIPUS:* ↑BDNF, ↑GDNF, ↑VEGF,
*in vitro* 84 astrocytes: CTX TNA2 LIPUS	Evaluation at 4, 8, 12, 24 h post-LIPUS	*f_c_:* 1 MHz *f_R_:* 10 Hz *I_SPTA_:* 110 mW/cm^2^ *Duration:* 15 min (3x5/5 on/off) *Duty cycle:* 50% *Burst length:* 50 ms *Transducer*: plane	*LIPUS:* ↑BDNF, ↑p-TrkB/TrkB, ↑p-Akt/Akt, ↑Ca^2+^ influx, ↓p-CREB/CREB, ↑p-NF-kB p65/NF-kB p65, ↑p-p65/p65
*in vitro* 76 preosteoblasts: MC3T3-E1 LIPUS	mRNA measurement 0, 6, 24, 48h post-LIPUS	*f_c_:* 1.5 MHz *f_R_:* 1 kHz *I_SATA_:* 30 mW/cm^2^ *Duration:* 20 min *Duty cycle:* 25% *Burst length:* 0.2 s *Transducer*: N/A	*LIPUS:* ↑CD200 (mRNA) at 24h, ↑bone morphology genes
*in vitro* 190 osteoblasts: MC3T3-E1 LIPUS	LPS: 1 μg/mL LIPUS/sham simultaneous with LPS	*f_c_:* 1.5 MHz *f_R_:* 100 kHz *I_SATA_:* 30 mW/cm^2^ *Duration:* 2 h *Duty cycle:* N/A *Burst length:* 0.2 ms *Transducer*: N/A	*LPS:* ↑CCL2 (mRNA), ↑CXCL1 (mRNA), ↑ CXCL10 (mRNA), ↑RANKL (mRNA), ↑NF-kB (mRNA) *LPS+LIPUS:* - CCL2 (mRNA), ↓CXCL1 (mRNA), ↓CXCL10 (mRNA), ↓RANKL (mRNA), ↓NF-kB (mRNA), ↓ERK/Akt/IKK/IRF3, ↓TLR4/MyD88 association
*in vitro* 114 endothelial: ECV 304	Applied laminar fluid shear stress to endothelial cell line	*Laminar fluid shear stress:* 15 dynes/cm^2^ (1.6 Pa) *Duration:* 5, 15, 30, 60 mins	*Shear Stress:* prolonged shear stress led to increased association between eNOS and HSP90, but did not lead to an increase in HSP90
*in vivo* 77 Female Sprague-Dawley rats LIPUS	6-OHDA: 12μg 2μL of 6-OHDA injected into right striatum 6-OHDA model of Parkinson's Disease Cellular evaluation at 8 weeks post-injection	*f_c_:* 1 MHz *f_R_:* 1 Hz *I_SPTA_:* 528 mW/cm^2^ *Duration:* 15 min (3x5/5 on/off) *Duty cycle:* 5% *Burst length:* 50 ms *Transducer*: focused, single hemisphere	*6-OHDA+LIPUS:* ↓GFAP+ astrocytes, ↓Iba1+ microglia, ↓IL-1b, ↓p-NF-kB, ↑GDNF, - BDNF (insignificant increase), ↑BBB stability (ZO-1, claudin-5) LIPUS started 2 weeks following 6-OHDA injection, LIPUS administered 5 days/week for next 6 weeks Observed no tissue damage with LIPUS regimen
*in vivo* 97 Beagles (2-8 years old) LIPUS	Wound healing in mucoperiosteal flap surgery	*f_c_:* 1.5 MHz *f_R_:* 1 kHz *I:* 30 mW/cm^2^ *Duration:* 20 min/day *Duty cycle:* N/A *Burst length:* 0.2 ms *Transducer*: 13 mm diameter	*LIPUS:* ↑HSP70 expression in gingival tissues (absent in control), ↑cementum and mandibular bone regeneration LIPUS continued for period of 4 weeks following surgery
*in vivo* 78 Female C57BL/6 mice (7 weeks old) LIPUS	LIPUS in model of repetitive restraint stress (RRS): 22 days LIPUS in cuprizone (0.2% in chow) model of demyelination: 35 days Analysis 24 hours following last LIPUS treatment	*f_c_:* 1.5 MHz *f_R_:* 1 kHz *I_SPTA_:* 25 mW/cm^2^ *Duration:* 20 min/day *Duty cycle:* 20% *Burst length:* N/A *Transducer*: plane	*LIPUS:* ↑BDNF (hippocampus) *RRS+LIPUS:* ↓anhedonia, ↑BDNF (hippocampus) *CUPRIZONE+LIPUS:* ↑MBP (myelin basic protein, cortex), ↑NG2 (neural/glial antigen 2, cortex) NG2 expressed in oligodendrocyte progenitors, and microglia
*in vivo* 79 Male C57BL/6 mice (8 weeks old) LIPUS	LPS: 250 μg/kg LPS administration from days 5-11 LIPUS treatment from days 7-13 Biological analysis at 4 (GFAP, Iba1, BDNF, CREB) and 48 h post-LIPUS	*f_c_:* 1 MHz *f_R_:* 1 Hz *I_SPTA_:* 528 mW/cm^2^ *Duration:* 15 min (3x5/5 on/off) *Duty cycle:* 5% *Burst length:* N/A *Transducer*: focal, (*D*: 38 mm, *ROC*:63.5 mm; half-max pressure amplitude of focal zone - *D:* 3 mm, *L:* 26 mm)	*LPS+LIPUS:* ↑spatial learning and memory (Morris water maze, novel object recognition), ↓Aβ (hippocampus, but not cortex), ↓cleaved caspase-3, ↓GFAP, - Iba1 (non-significant decrease), ↓TNFa, ↓IL-1b, ↓IL-6, ↓p-NF-kB, ↓TLR4, ↑BDNF, ↑p-CREB *LIPUS:* ↑BDNF, ↑p-CREB, ↑p-NF-kB (cortex), ↑TLR4 Increase in TLR4 and p-NF-kB in LIPUS group did not correspond to a significant increase in TNFa, IL-1b, or IL-6
*in vivo* 64 *Vascular Dementia* Male C57BL/6 mice (10-12 weeks old), eNOS-/- (C57BL/6 background) *Alzheimer's Disease* Male 5XFAD transgenic mice (14-16 weeks old, C57BL/6 background) LIPUS	LIPUS applied every other day following surgery (i.e. 1, 3, 5; BCAS or sham for vascular dementia) LIPUS applied on days: 1, 3, 5, 28, 30, 32, 56, 58, 60, 84, 86 for AD model, testing on day 86	*f_c_:* 1.875 MHz *f_R_:* 6 kHz *I_SPTA_:* 90 mW/cm^2^ *Duration:* 20 min (x3: right, midline, left)/day *Duty cycle:* 5% *Burst length:* 17μs *Transducer*: focal, (*focus:* 9 cm, *beam width:* 3.6-4 mm)	*VaD+LIPUS - Molecular (day 3/7):* ↓WM lesions, ↑angiogenesis and OPC genes (mRNA), ↑eNOS (both inactivated and activated; correlated with increased NGF, BDNF, VEGF), ↑GFAP, ↑Olig2^+^ *VaD+LIPUS - Behavioural (day 28):* ↑cognitive function (Y-maze, passive avoidance, novel object recognition, not all significant) *VaD+LIPUS - CBF:* ↑CBF with LIPUS over time (start at day 2, continues to day 28) *VaD+LIPUS+eNOS^-/-^ (with respect to VaD):* - behavioural, - CBF, - WM lesions, - NGF, - BDNF, - VEGF *5XFAD+LIPUS:* ↑HSP90, ↑CBF, ↑immune and angiogenesis genes (mRNA), ↑cognitive function (Y-maze), ↓Iba1+, ↑eNOS, ↑BDNF, ↓Aβ Note: HSP90 not measured in VaD group, only 5XFAD
*in vivo* 80 Male C57BL/6 mice (8 weeks old) LIPUS	Cortical Impact Injury (CCI) - model of traumatic brain injury (TBI) LIPUS applied 5 mins post-CCI, daily thereafter Analysis at day 1, 4, and 148	*f_c_:* 1 MHz *f_R_:* N/A *I_SPTA_:* 528 mW/cm^2^ *Duration:* 5 min/day *Duty cycle:* N/A *Burst length:* N/A *Transducer*: focal, (*focal diameter:* 3 mm, *focal width:* 26 mm, *diameter:* 38 mm, *ROC:* 63.5 mm)	*TBI+LIPUS (day 1):* -MMP-9, ↓neutrophils, ↓Iba1+, -p-Foxo1, ↓neurological deficits *TBI+LIPUS (day 4):* ↓MMP-9, ↓neutrophils, ↓Iba1+, ↑p-Foxo1, ↓neurological deficits *TBI+LIPUS (day 148):* insignificant ↑BDNF+ neurons, ↑neurons, ↓T2w edema volume, ↓neurological deficits

## Abbreviations

CNS: central nervous system
BBB: blood brain barrier
HSPs: heat shock proteins
LIPUS: low intensity pulsed ultrasound
HSR: heat shock response
MRgFUS: magnetic resonance guided focused ultrasound
ET: Essential tremor
HIFU: high intensity focused ultrasound
SRS: stereotactic radiosurgery
T2w: T2-weighted
MR: magnetic resonance
MHC: major histocompatibility complex
APC: antigen presenting cell
CD: cluster of differentiation
DC: dendritic cell
EV: extracellular vesicle
miRNA: microRNA
IL: interleukin
DAMP: damage associated molecular pattern
PAMP: pathogen associated molecular pattern
PRR: pattern recognition receptor
TLR: toll like receptor
DNA: deoxyribonucleic acid
RNA: ribonucleic acid
TIR: toll/interleukin-1 receptor
MyD88: myeloid differentiation primary response 88
MAL: MyD88 adaptor like
IRAK: interleukin-1 receptor-associated kinase
TRAF: tumour necrosis factor receptor-associated factor
JNK: c-Jun N-terminal kinase
NF-κB: nuclear factor kappa B
IκB: inhibitor of NF-κB
IKK: IκB kinase
TNF: tumour necrosis factor
IRF: interferon regulatory factor
IFN: interferon
TRIF: TIR domain containing adaptor-inducing interferon beta
LPS: lipopolysaccharide
SN: substantia nigra
TNFR: TNFα receptor
mRNA: messenger RNA
CD200R: CD200 receptor
Aβ: amyloid beta
IFNAR: type I IFN receptor
HMGB1: high mobility group box protein 1
AD: Alzheimer's disease
CSF: cerebral spinal fluid
ROS: reactive oxygen species
NOD: nucleotide-binding oligomerization domain
NLRP3: NOD-like receptor family pyrin domain containing 3
FD: female derived
TREM2: triggering receptor expressed on myeloid-2
MD: male derived
eNOS: endothelial nitric oxide synthase
CBF: cerebral blood flow
E2: 17β-estradiol
ERα: estrogen receptor α
BDNF: brain derived neurotrophic factor
*I_SPTA_*: spatial peak temporal average intensity
*f_R_*: pulse repetition frequency
CREB: cyclic adenosine monophosphate response element-binding protein
Akt: protein kinase B
TrkB: tyrosine protein kinase B
TBI: traumatic brain injury
MMP-9: metalloproteinase 9
FOXO1: forkhead box protein O1
6-OHDA: 6-hydroxydopamine
PD: Parkinson's disease
GDNF: glial cell line-derived neurotrophic factor
VaD: vascular dementia
Cav-1: caveolin-1
HSP: heat shock protein
EAE: experimental autoimmune encephalomyelitis
iNOS: inducible nitric oxide synthase
AMP: adenosine monophosphate
AMPK: AMP activated protein kinase
mTOR: mammalian target of rapamycin
ERK: extracellular signal-regulated kinase
PBMC: peripheral blood mononuclear cell
Dok2: docking protein 2
RasGAP: Ras GTPase activating protein
IP-10: IFNγ-inducible protein 10
MCP-1: monocyte chemoattractant protein-1
HSF1: heat shock factor 1
HSE: heat shock element
PI3K: phosphatidylinositol-3-kinase
MAPK: mitogen-activated protein kinase
Sirt1: sirtuin-1
HIF1α: hypoxia inducible factor 1α
CHBF: constitutive heat shock element binding factor
dsDNA: double strand DNA
Pol II: RNA polymerase II
NRF2: nuclear factor erythroid 2-related factor 2
IRG1: immune responsive gene 1
HUS: high intensity ultrasound
TNFAIP3/A20: TNFα-induced protein 3
ARE: antioxidant response element
Apaf-1: apoptotic protease-activating factor 1
ES: electric stimulation
SOCS: suppressor of cytokine signalling 3
